# Hepatitis C mortality trends in San Francisco: can we reach elimination targets?

**DOI:** 10.1016/j.annepidem.2021.10.004

**Published:** 2021-10-23

**Authors:** Ali Mirzazadeh, Shelley N. Facente, Katie Burk, James G. Kahn, Meghan D. Morris

**Affiliations:** a Department of Epidemiology and Biostatistics, University of California, San Francisco; b Institute for Global Health Sciences, University of California, San Francisco; c Facente Consulting, San Francisco, CA; d Community Health Equity and Promotion Branch, San Francisco Department of Public Health, San Francisco, CA; e Philip R Lee Institute for Health Policy Studies, University of California San Francisco, San Francisco; f School of Public Health, University of California, Berkeley

**Keywords:** Hepatitis c, Mortality, Hcv elimination targets

## Abstract

**Purpose:**

Hepatitis C virus (HCV) is the most common blood-borne infection in the United States, and a leading cause of liver disease, transplant, and mortality. CDC HCV elimination goals include reducing HCV-related mortality by 65% (from 2015) by 2030.

**Methods:**

We used vital registry data (CDC WONDER) to estimate overall and demographic-specific HCV-related mortality from 1999 to 2019 in San Francisco and then used an exponential model to project progress toward HCV elimination. Local trends were compared to state and national trends.

**Results:**

Between 1999 and 2019, there were 1819 HCV-related deaths in San Francisco, representing an overall age-adjusted mortality rate of 9.4 (95% CI 9.0, 9.9) per 10 0,0 0 0 population. The age-adjusted HCV-related mortality rates were significantly higher among males (13.7), persons aged 55 years and older (28.0), Black and/or African Americans (32.2) compared to other racial groups, and Hispanic/Latinos (11.6) compared to non-Hispanic and/or Latinos. Overall and in most subgroups, mortality rates were lowest between 2015 and 2019. Since 2015, San Francisco observed a significantly larger reduction in agbe-adjusted HCV-related mortality than California or the U.S. Projected age-adjusted HCV-related mortality rates for San Francisco for 2020 and 2030 were 4.7 (95% CI 3.5, 6.2) and 1.1 (95% CI 0.7, 1.8), respectively.

**Conclusions:**

Based on trends between 2015 and 2019, San Francisco, California, and the U.S. are projected to achieve 65% reduction in HCV-mortality at or before 2030. Based on current trends, San Francisco is projected to achieve this goal earlier.

## Introduction

Hepatitis C virus (HCV) is the most common blood-borne infection in the United States [1], with an estimated 2.4 million people (1% of all adults) living with chronic HCV infection [2]. Nationally, in 2018 there were 50,300 people newly infected with hepatitis C [3], with young adults and people who inject drugs experiencing the highest rates of new HCV infections [3, 4]. In late 2014, the first all-oral direct-acting antiviral (DAA) agents for treating HCV were approved by the U.S. Food and Drug Administration (FDA) and proved to be a game-changer for HCV treatment compared with previous interferon-based therapies. In 2016, the FDA approved pangenotypic regimens, which further simplified and increased the accessibility of HCV treatments. Increased market competition and improved insurance reimbursement significantly reduced price-related barriers and further improved access.

In 2018, the CDC’s National Center for Health Statistics reported 15,713 HCV-related deaths, corresponding to 3.72 deaths per 100,000 standard population in the United States [3]. HCV-related mortality is the highest in Western and Southern regions of the U.S., including the state of California where HCV death rate is higher than the US rate (death rate ratio 1.35) [5]. The advent of curative DAA therapies prompted the World Health Organization (WHO) and Centers for Disease Control and Prevention (CDC) to target HCV elimination by 2030. In addition to reducing the number of new HCV infections and increasing the number of people treated with DAAs, progress toward elimination includes a 65% reduction (from 2015) in HCV-related mortality by 2030.

Micro-elimination efforts are necessary to achieve national and global elimination goals [6]. In 2016, San Francisco was the first U.S. county to establish a local initiative to eliminate HCV [7]. End Hep C SF is a collective impact initiative aimed at eliminating the public health burden of HCV by increasing prevention, testing and linkage, and treatment services for communities hardest hit by HCV [8].

This study relied on vital registry data between 1999 and 2019 to assess changes in HCV-related mortality trends pre and post DAA treatment availability. We examined HCV-related mortality trends at the local (county) level and estimated progress toward achieving a 65% reduction (from 2015) in HCV-related mortality by 2030. To assess the impact of micro-elimination efforts, we compared San Francisco trends to those at the state and national levels.

## Materials and methods

For this analysis, we used publicly available mortality data from the CDC Wide-ranging ONline Data for Epidemiologic Research (CDC WONDER) [9]. We used multiple cause of death (detailed mortality) data for deaths reported between 1999 and 2019 coded using the tenth revision of the International Classification of Diseases (ICD-10). The following codes inform our definition as recommended by the National Center for Health Statistics (NCHS) [10]: HCV-related mortality: B17.1 (acute hepatitis C) or B.18.2 (chronic hepatitis C).

We report HCV-related mortality within demographic subgroups supplied within CDC-WONDER, defined by sex (female, male), age groups (under 25 years, 25–54 years, 55 years and over), baby boomers (people born between 1945 and 1965), race (American Indian or Alaska Native, Asian or Pacific Islander, Black or African American, White), Hispanic origin (Hispanic or Latino, Not Hispanic or Latino, Not Stated). Hispanics and/or Latinos also includes whites and people of other races. To assess the annual trend in mortality rates in baby boomers birth cohort, we reported HCV-related mortality for baby boomers between 1999 and 2019.

## Analysis

First, to assess trends in overall and demographic-specific HCV-related mortality at the San Francisco County level we calculated the mortality age-adjusted rates using direct standardization based on the 20 0 0 standard population with 95% confidence intervals (95% CI) and standard errors (SE). In years with 100 or more deaths, SE was calculated as the death rate times the square root of one over thenumber of deaths; in years with 99 or fewer deaths, SE was calculated as the standard error of a Poisson distribution of the number of deaths [11].

To assess the trend in HCV-related mortality overall and in subgroups while avoiding issues with sparse data, we combined mortality data into 5 year periods (1999–2004, 2005–2009, 2010–2014, 2015–2019). We compared the HCV-related mortality rates for 2015–2019 to earlier periods (i.e., 1999–2014) using a random-effects meta-regression model, because 2015 was the first full year in which DAA therapies were available. We also assessed the differences in HCV-related mortality rates between subgroups using random-effects meta-regression models. The meta-regression coefficients (B: Beta) showed change in HCV-related mortality between the two time periods (1999–2014 vs. 2015–2019).

Lastly, we assessed progress toward WHO elimination goals for mortality by applying age-adjusted HCV-related mortality rates (point, lower and upper estimates) from 2015 to 2019 to fit three exponential decay models: one on point estimates, one on lower estimates and one on upper estimates:

$$N_{t}=N_{0}e^{-rt}$$

- *t* = time 0,1 2, … (year since 2015)
- N_0_ = Starting value (number of deaths in 2015)
- *e* = Euler’s constant (2.71828)
- N_t_ = Number of deaths at year t
- *r* = the rate of decay (when *r <* 0, the number of deaths exponentially declines)

The fit for all models was assessed by R^2^; it was more than 0.81 for all models indicating good fit. We compared analyses at the San Francisco level to those at the state level for California and national level for the United States. We used freely available R 3.6.3 software (R Core Team, Vienna, Austria) for all analysis.

We did a sensitivity analysis to assess whether higher uncertainty in future projection may affect our conclusion on reaching 2020 and 2030 elimination targets; we increased the projections’ errors (i.e., SE) per year with a scale of 0% to 30% and recalculated the lower limits and upper limits of the 95% CI for projections for San Francisco, California, and the United States. Sensitivity analysis results are presented in Supplemental Figures 1 to 3.

## Results

### Demographic trends in HCV-related deaths in San Francisco

Between 1999 and 2019 (Table 1), there were 1819 HCV-related deaths in San Francisco County, representing an overall age-adjusted HCV-related mortality rate of 9.4 (95% CI 9.0, 9.9) per 10 0,0 0 0 standard population. The age-adjusted HCV-related mortality rates were significantly higher among males versus females (13.7 vs. 5.2, *P* =.001) and people aged 55 years and older versus 25–54 years old (28.0 vs. 8.0, *P* =.001). Both Whites and Blacks/African Americans had higher HCV-related mortality compared to Asians and/or Pacific Islanders (32.2 and 11.5 vs. 2.2, *P* =.001), and HCV-related mortality was higher among Hispanics and/or Latinos (11.6) versus non-Hispanics and/or Latinos (8.9, *P* =.001).

Age-adjusted HCV-related mortality decreased from between 9.8 and 11.0 deaths per 100,000 standard population during 1999–2014 to 7.4 (6.7 – 8.1) per 100,000 during 2015–2019 (*P* =.074) (Table 1). A statistically significant temporal reduction in HCV-related mortality occurred among males (*B* = −3.3, *P* =.044), and non-Hispanics (*B* = −2.8, *P* =.037) between the two time periods. The largest reduction was observed among persons 25–54 years old (*B* = −5.9, *P* =.093).

### San Francisco HCV-related mortality in baby boomers birth cohort

**Fig. 1.**
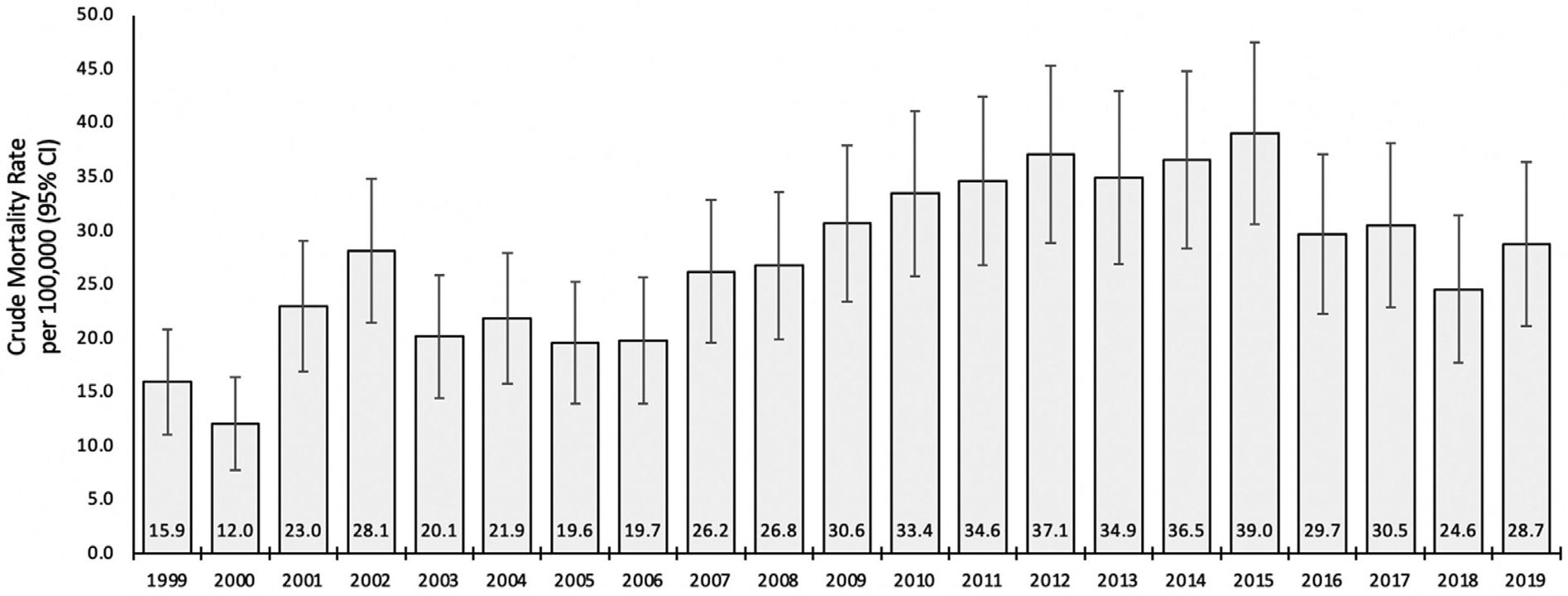
San Francisco County Hepatitis C Mortality Rates for Baby Boomers Birth Cohort (People born 1945–1965) by year.

We examined annual HCV-related mortality rates in San Francisco baby boomers from 1999 to 2019 (Fig. 1). HCV-related mortality rate in San Francisco baby boomers increased from 15.9 (95% CI 11.0 – 20.8) in 1999 to 39.0 (95% CI 30.6 – 47.5) per 100,000 population in 2015. There were fewer deaths per 100,000 population in baby boomers from 2016 to 2019 (ranged 24.6 to 30.5) than what was reported for 2015 (i.e., 39.0).

### Trends and elimination target projections in San Francisco, California, and the United States

**Fig. 2.**
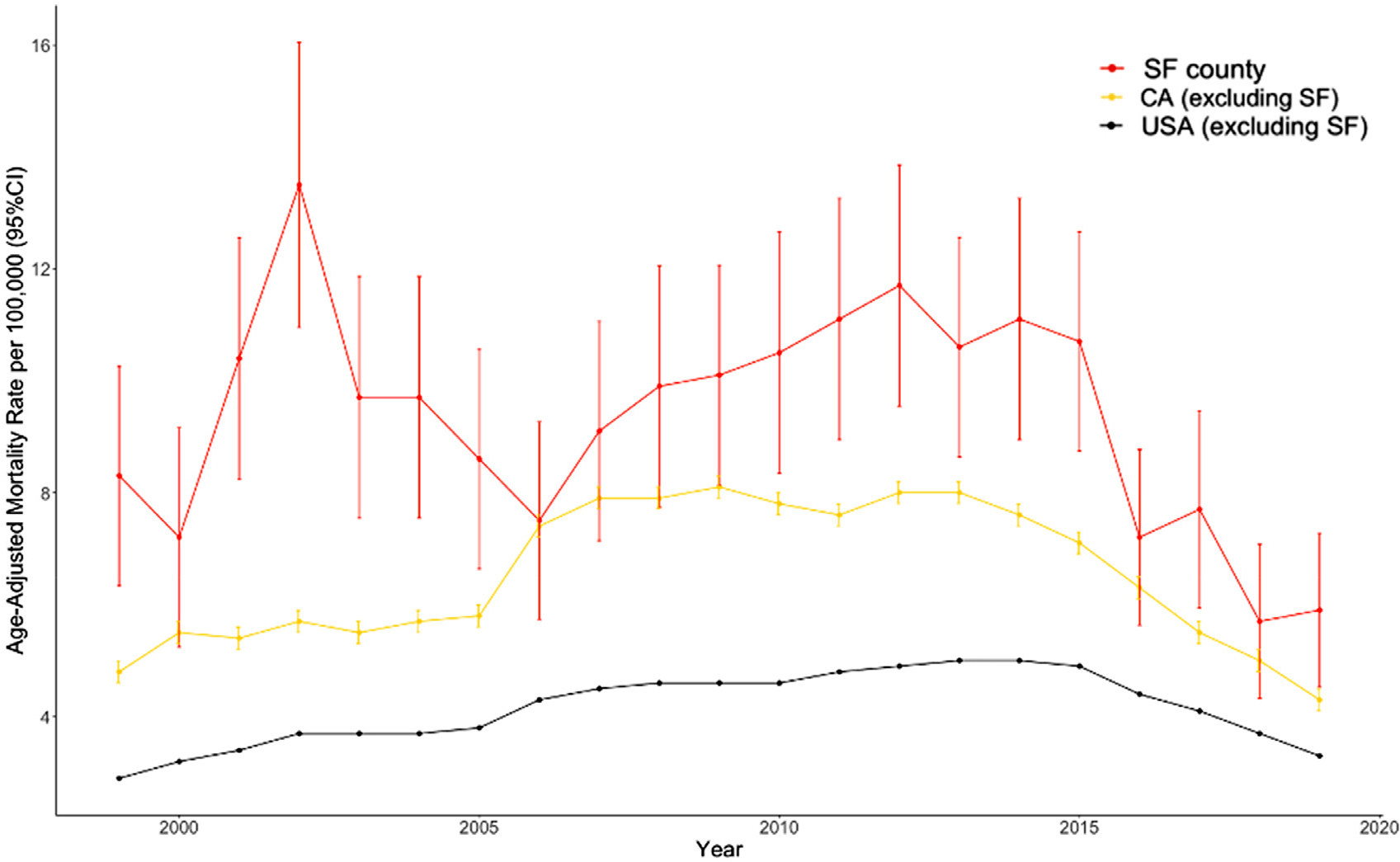
Hepatitis C mortality in San Francisco, California, and the United States, 1999 to 2019.

**Fig. 3.**
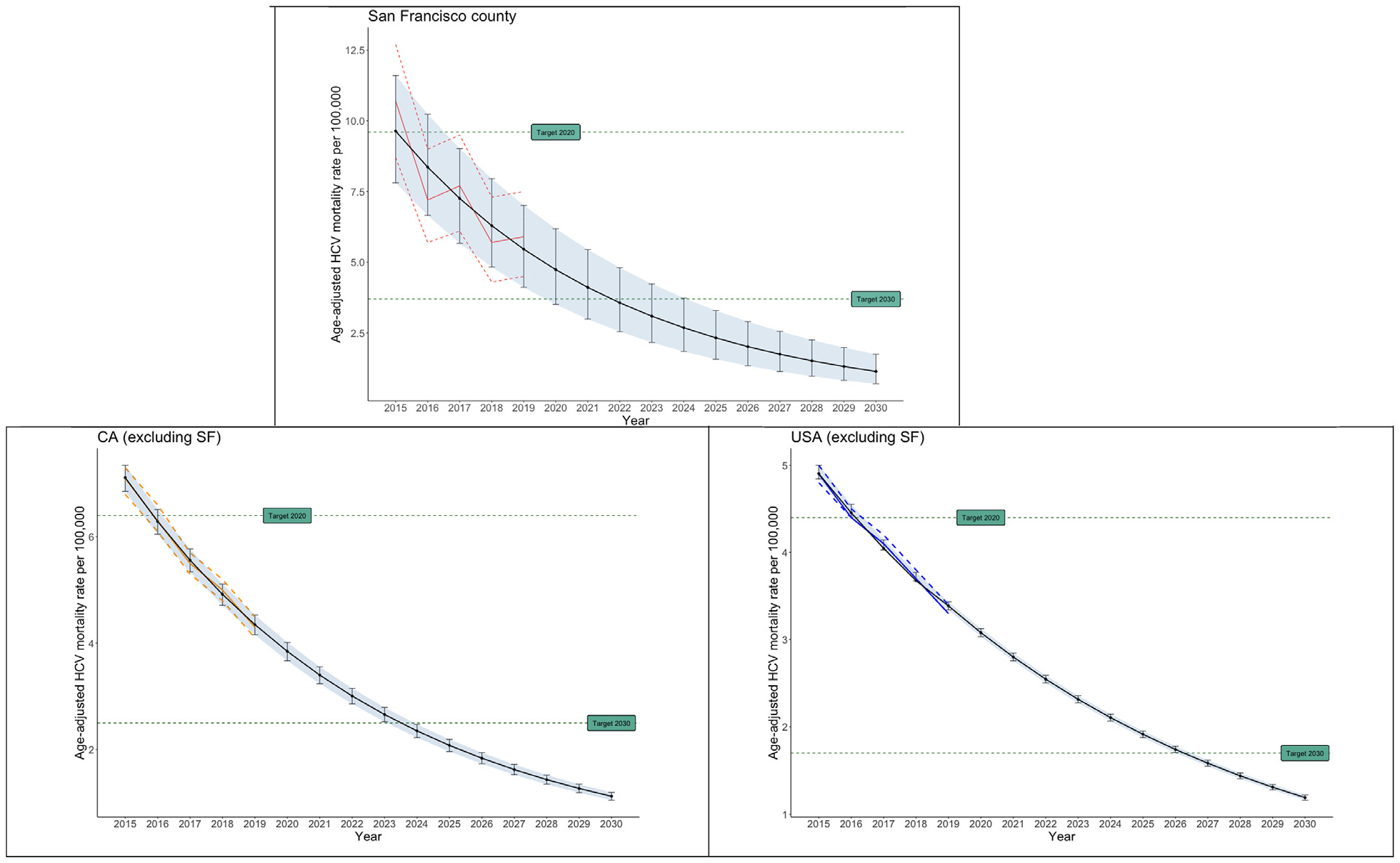
Projection of age-adjusted HCV-related mortality in San Francisco, California and the United States till 2030 against elimination targets. Footnote: Observed data (point estimates, lower and upper limits: red color) in 2015–2019 were projected till 2030 using exponential models. The elimination target for 2020 was a 10% reduction in HCV-related mortality observed for 2015 and the elimination target for 2030 was a 65% reduction in HCV-related mortality observed for 2015.

Over the entire period from 1999 to 2015, annual age-adjusted HCV-related mortality in San Francisco County was significantly higher than the corresponding annual rates in California and the United States (Fig. 2). The projected age-adjusted HCV-related mortality rates for San Francisco County were 4.7 (95% CI 3.5, 6.2) for 2020 and 1.1 (95% CI 0.7, 1.8) for 2030 (Fig. 3). The projected age-adjusted HCV-related mortality rates for the state of California were 3.8 (95% CI 3.7, 4.0) for 2020 and 1.1 (95% CI 1.1, 1.2) for 2030. The projected age-adjusted HCV-related mortality rates for the United States were 3.0 (95% CI 3.0, 3.1) for 2020 and 1.2 (95% CI 1.2, 1.3) for 2030.

### Sensitivity analysis

Sensitivity analysis results are presented in Supplemental Figures 1 to 3. The projected age-adjusted HCV-related mortality rates for San Francisco were significantly lower (2018: 6.3, 95% CI 4.8, 8.0) than the elimination target in 2020 even after increasing the error by 25% per year, and were significantly lower (2030: 1.1, 95% CI 0.7, 1.8) than the elimination target in 2030 even after increasing the error by 20% per year. The projected age-adjusted HCV-related mortality rates for California and the United States were significantly lower than the elimination targets in 2020 and 2030 even after increasing the error by 30% per year.

## Discussion

Our findings suggest that San Francisco, California, and the United States have reached the WHO HCV-related mortality elimination targets for 2020 (10% reduction from 2015) and are projected to reach the targets for 2030 (65% reduction from 2015) assuming continuation of decreasing mortality trends observed between 2015 and 2019. This analysis illustrates how available data can be harnessed to assess progress toward HCV elimination goals in a local area, to help assess local intervention strategies.

Projecting 2015–2019 annual mortality rates to 2030 showed that it is feasible for San Francisco to reach WHO’s HCV mortality elimination targets. San Francisco may achieve the 2030 elimination targets as early as 2023. Furthermore, it is likely that the impacts of End Hep C SF’s diagnosis and treatment interventions have not been realized, rendering these projection models conservative. However, the COVID-19 pandemic disrupted health services, with documented impact on HCV care and treatment in the United States and worldwide [12], particularly for African Americans, who are disproportionately impacted by both COVID-19 and HCV. Further, San Francisco has may have more work to do to meet the WHO elimination goals for HCV incidence and diagnosis; reaching mortality targets is only one component of success. Our findings suggest that California may reach the elimination target for HCV mortality, similar to what reported by Sulkowski et al. [13]. Sulkowski et al. estimated 2039 as the year in which California may reach all elimination targets for HCV (for incidence by 2039, for mortality by 2020, for diagnosis by 2030, and for treatment by 2035).

HCV-related mortality has significantly declined in San Francisco since 2015. We found San Francisco HCV mortality rates (per 100,000 standard population) decreased from 11.0 in 2010–14 to 7.4 in 2015–19; a decreasing patten that also reported by Hall et al. model [14] with −10.40 average annual percent change from 2013 to 2017. Though there has been a multi-level HCV elimination initiative active in San Francisco since DAAs first became available in 2015, it is unlikely that we would already be able to detect a reduction in HCV-related mortality solely because of these interventions. There are several other possible explanations for this reduction. First, there is competing mortality risk: deaths related to drug and alcohol use have increased significantly in San Francisco since 2016 [15]. As more people die from drug overdose and alcohol use (including people living with HCV), this reduces the number with potential to die from HCV infection, especially given that people who inject drugs account for 68% of the 12,300 people estimated to live with HCV in San Francisco [16]. Second, baby boomers once comprised a majority of people with chronic HCV in San Francisco, but in recent years the proportion of people living with HCV who are young has substantially increased. As it typically takes 20–30 years for a person with untreated HCV to die from HCV-related complications [17], it is reasonable to assume that as the average age of people living with HCV in San Francisco decreases, the overall HCV-related mortality rate would also decrease.

San Francisco has had a greater drop in annual age-adjusted mortality rates than those observed in California and nationally. The difference in HCV mortality trends can potentially be explained by a number of characteristics that make San Francisco unique. Since before the Affordable Care Act, San Francisco offered Healthy San Francisco, which provides no-cost health care to adults who are otherwise uninsured. San Francisco also has a very high median income (ranking 13 of 24,011 cities in the United States) [18]; strong academic medical centers with expertise treating HCV, including among people who inject drugs; and younger residents (rank 8721 of 15,354 cities in the United States) [19] who can afford the high costs of living in the San Francisco Bay Area. Local initiatives like End Hep C SF [7], which have engaged health providers and communities to address HCV more efficiently by increasing community-based HCV testing and training primary care providers to treat HCV in primary care and non-traditional settings (such as drug treatment programs), may have at least played a contributing role in reducing HCV-related mortality more quickly in San Francisco compared to California and the United States.

We observed disparities in HCV-related mortality across racial and ethnic groups. While the acute HCV infection rate per 100,000 population is 1.3 among Whites and 0.6 among Blacks and/or African Americans in the US [20], the HCV-related *mortality* among Blacks and/or African American has consistently been about three times higher than Whites since 2005. Systemic racism in health care access and delivery is well documented, leading to reduced health care access for Blacks and/or African Americans, including for HCV diagnosis and treatment [21]. Related, disparities exist in prevalence of chronic stress and associated comorbidities among Blacks and/or African Americans compared to other races and/or ethnicities, including cardiovascular disease [22], alcohol-related cirrhosis [23], and smoking [24], which could potentially explain higher HCV mortality risk among Blacks and/or African Americans due to accelerated liver damage caused by these co-morbidities.

We used exponential decay models for future projections of HCV mortality. The exponential decay model has two parameters (N_0_: starting value, r: the rate of decay) to be estimated by the regression method based on historical data (e.g., 2015 to 2019 in our model). Although all our models had a good fit (R^2^ > 0.81), the future projections assume the trend between 2015 and 2019 to be continued. Future decline in HCV screening and treatment efforts or decrease in HCV burden in coming years that makes it more difficult and costly to identify and treat remaining infections may slow the rate of decline in HCV mortality. The uncertainty in future projections is another limitation of our model, and we assessed its impact on our conclusions by a sensitivity analysis. We increased the projections’ errors (i.e., SE) per year with a scale of 0% to 30% and recalculated the lower limits and upper limits of the 95% CI for projections. Sensitivity analysis results suggested San Francisco would reach elimination targets for 2030 even after increasing the error by 20% per year, and California, and the United States to reach elimination targets for 2030 even after increasing the error by 30% per year.

Our study has at least three important limitations. First, demographic data such as race were usually collected by an informant or relative of the deceased and so are subject to misclassification. Second, causes of deaths were collected based on the report of physicians or medical examiners; since HCV is initially asymptomatic and can remain undiagnosed, it is underreported as cause for mortality [25–27]. Third, we were unable to examine individual-level data for demographic, comorbidities, and risk factors for HCV mortality, and therefore could assess only aggregate trends available through CDC-WONDER.

Our study found progress locally, statewide, and nationally towards the WHO HCV elimination mortality goal, but also identified subpopulations (men, those aged 55 years and older, Blacks and/or African Americans, and Hispanics and/or Latinos) who were at increased risk for HCV-related mortality in San Francisco. Improving linkage to HCV cure for these priority subgroups will be needed to achieve elimination targets across all populations and communities.

## Supplementary Material

Supplemental Figures

## Figures and Tables

**Table 1:** Age-adjusted Mortality Rate Associated with Hepatitis C in San Francisco County, 1999 to 2019

	Total (1999–2019)			1999–2004		2005–2009		2010–2014		2015–2019			Test 2015–19 vs. before,^‡^
	#	Rate* (95%CI)	P-value	#	Rate* (95%CI)	#	Rate* (95%CI)	#	Rate* (95%CI)	#	Rate* (95%CI)	P-value	Beta C. (P-value)
Overall	1819	9.4 (9.0 – 9.9)		481	9.8 (9.0 – 10.7)	402	9.1 (8.2 – 9.9)	539	11.0 (10.1 – 12.0)	397	7.4 (6.7 – 8.1)		*B* = −2.4 (0.074)
Sex													
Female	504	5.2 (4.7 – 5.6)	0.001	127	5.1 (4.2 – 6.0)	107	4.9 (3.9 – 5.8)	159	6.4 (5.4 – 7.4)	111	4 (3.3 – 4.8)	0.001	*B* = −1.3 (0.133)
Male	1315	13.7 (12.9 – 14.4)		354	14.5 (13.0 −16.0)	295	13.3 (11.7 – 14.8)	380	15.5 (14.0 −17.1)	286	10.8 (9.6 – 12.1)		*B* = −3.3 (0.044)
Age groups													
Under 25 years	Suppressed^†^	0.001									0.001		
25–54 years	605	8.0 (7.3 – 8.6)		261	12.3 (10.8 – 13.8)	152	8.6 (7.2 – 9.9)	134	7.3 (6.1 – 8.6)	58	3.1 (2.3 – 4.0)		*B* = −5.9 (0.093)
55 years and over	1212	28.0 (26.4 – 29.6)		218	20.9 (18.1 – 23.7)	250	25.0 (21.9 – 28.1)	405	36.8 (33.2 – 40.4)	339	28.4 (25.3 – 31.4)		*B* = 0.8 (0.923)
Baby Boomers^§^	1244	24.6 (23.1 – 25.9)		291	18.6 (16.4 – 20.8)	272	23.8 (20.9 – 26.6)	376	35.2 (31.7 – 38.8)	305	29.9 (26.5 – 33.3)		*B* = 4.6 (0.249)
Race													
American Indian or	Suppressed^†^	0.001									0.001		
Alaska Native													
Asian or Pacific	169	2.2 (1.8 – 2.5)		38	2.1 (1.5 – 2.9)	36	2.1 (1.4 – 2.9)	56	2.7 (2.1 – 3.6)	39	1.7 (1.2 – 2.3)		*B* = −0.5 (0.184)
Islander													
Black or African	468	32.2 (29.3 – 35.2)		117	28.3 (23.1 – 33.4)	103	29.3 (23.6 – 35.0)	149	41.8 (35.0 – 48.7)	99	27.3 (22.1 – 33.3)		*B* = −5.3 (0.482)
American													
White	1173	11.5 (10.9 – 12.2)		324	12.0 (10.7 – 13.3)	262	11.1 (9.7 – 12.4)	331	13.1 (11.7 – 14.5)	256	9.5 (8.3 – 10.6)		*B* = −2.3 (0.078)
Hispanic origin													
Hispanic or Latino	245	11.6 (10.1 – 13.0)	0.001	76	14.7 (11.6 – 18.4)	46	9.8 (7.1 – 13.1)	68	12.7 (9.8 – 16.1)	55	9.3 (7.0 – 12.2)	0.043	*B* = −2.6 (0.273)
Not Hispanic or	1527	8.9 (8.4 – 9.3)		401	9.2 (8.3 – 10.1)	356	9.0 (8.0 – 9.9)	460	10.5 (9.6 – 11.5)	310	6.5 (5.7 – 7.2)		*B* = −2.8 (0.037)
Latino													
Not Stated	47	NA		4	NA	0	NA	11	NA	32	NA		

NA = Not Applicable.

* Rates were per 10 0,0 0 0 and adjusted for age using 20 0 0 U.S. Std. Population;

† suppressed as the number was bellow ten;

‡ Beta coefficients from random-effects meta-regression were used to test for difference in rates for 2015–2019 vs. before;

§ Crude rates for Baby Boomers (Birth Cohort for people born 1945–1965) per 100,000.

## Data Availability

The data that were used in our analysis are Multiple Cause of Death which are available via www.cdcwoder. https://wonder.cdc.gov/controller/datarequest/D77
