# Supplementary material for: Hepatitis C mortality trends in San Francisco: can we reach elimination targets?
Source: Ann Epidemiol. Author manuscript; Available in PMC 2023 Jan 1. (PMC9293250; doi:10.1016/j.annepidem.2021.10.004)
Supplement: Supplemental Figures [file NIHMS1815498-supplement-Supplemental_Figures.docx]

| **Supplemental figure 1.** Sensitivity analysis on future projection of HCV mortality for San Francisco. The errors were increased per year for 0%, 5%, …, 30%. | 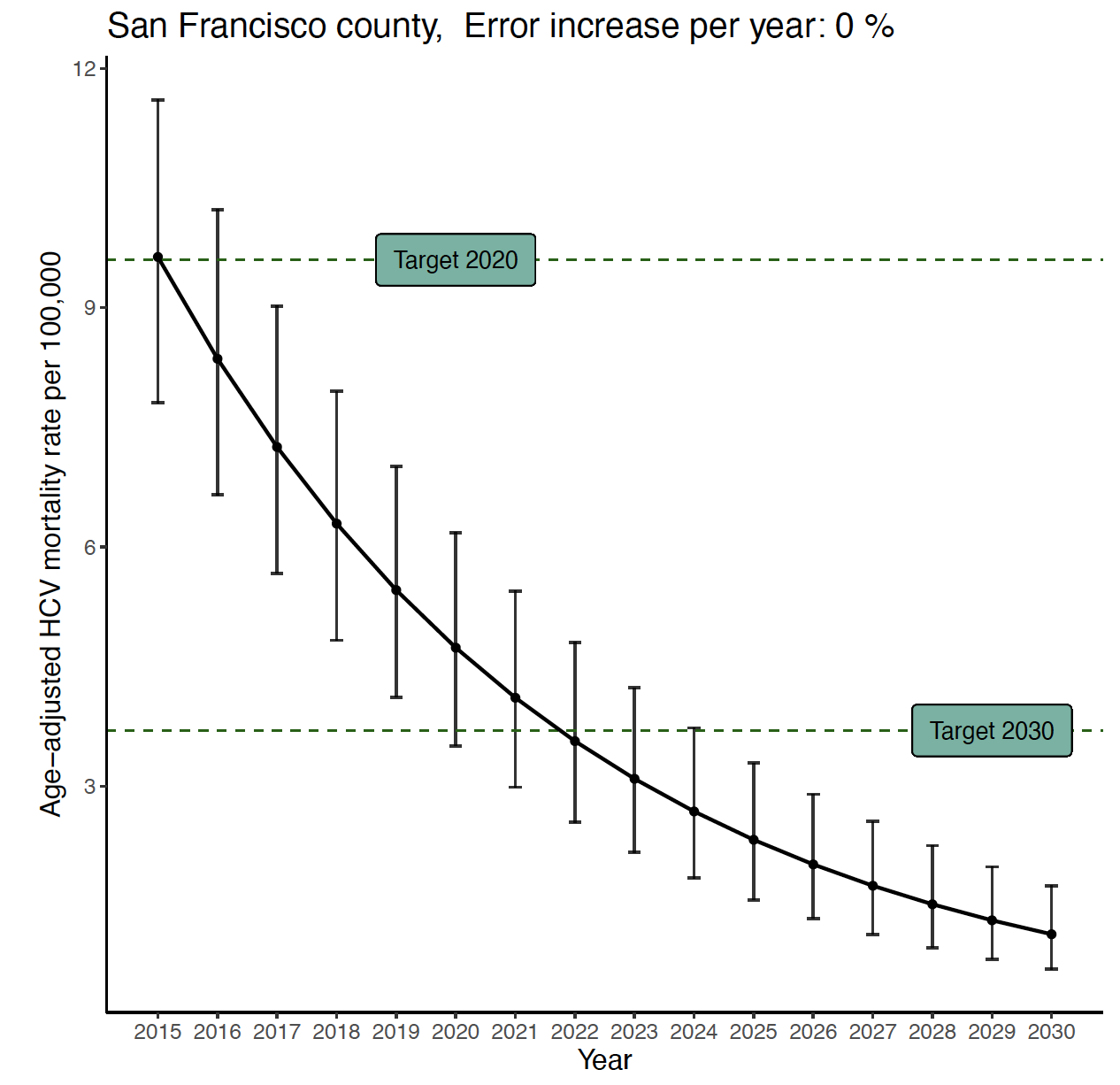 |
| --- | --- |
| 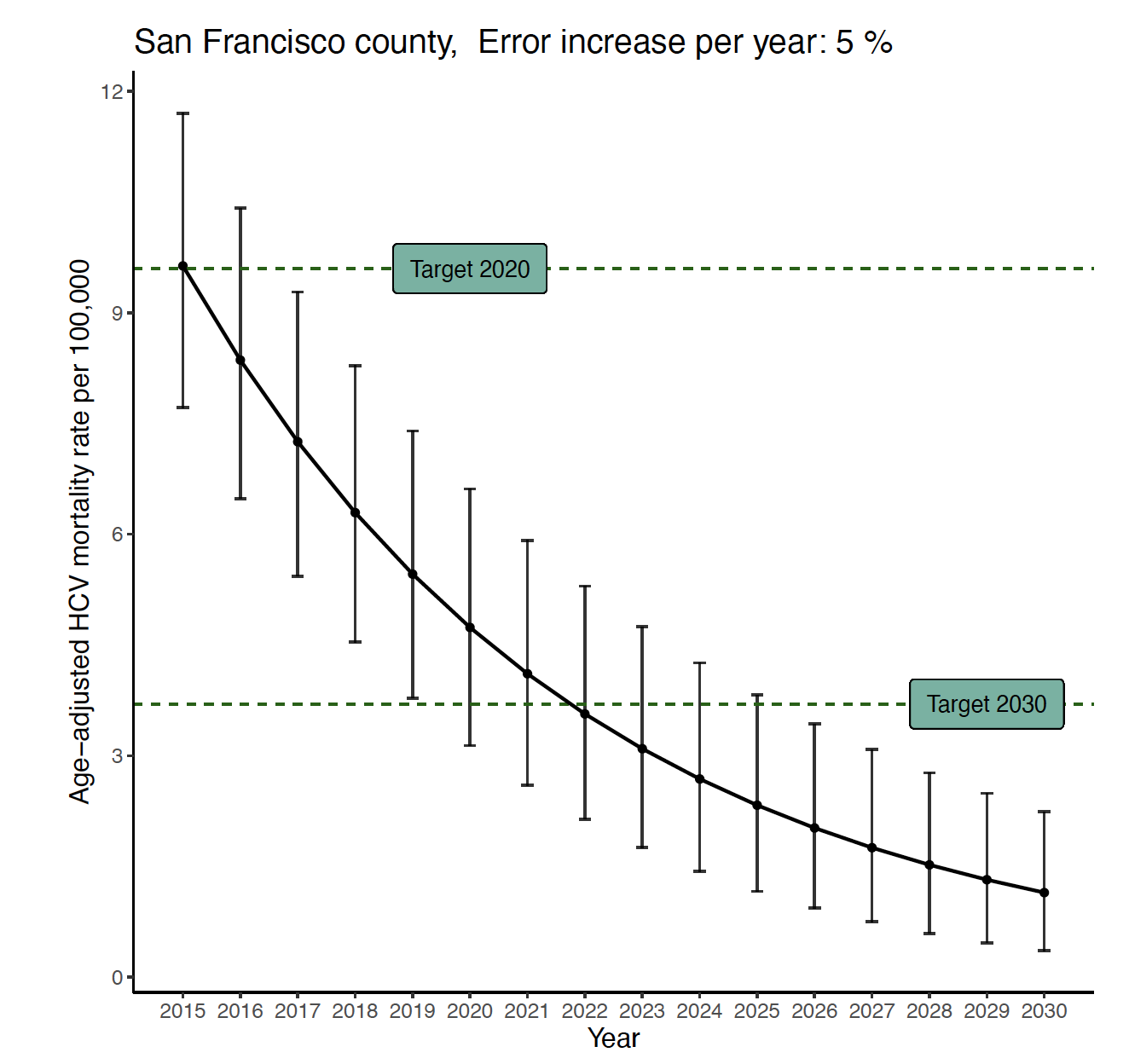 | 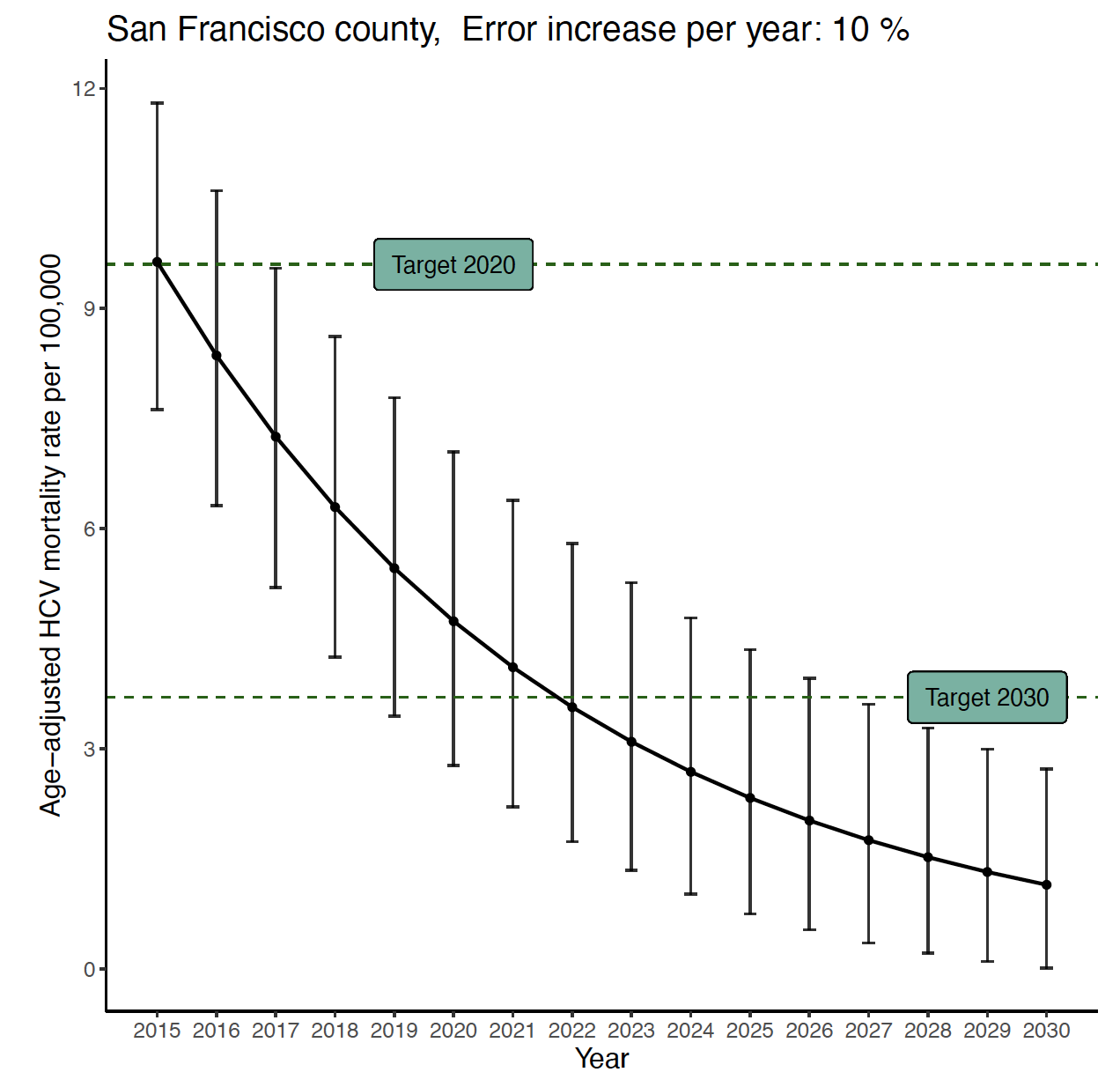 |
| 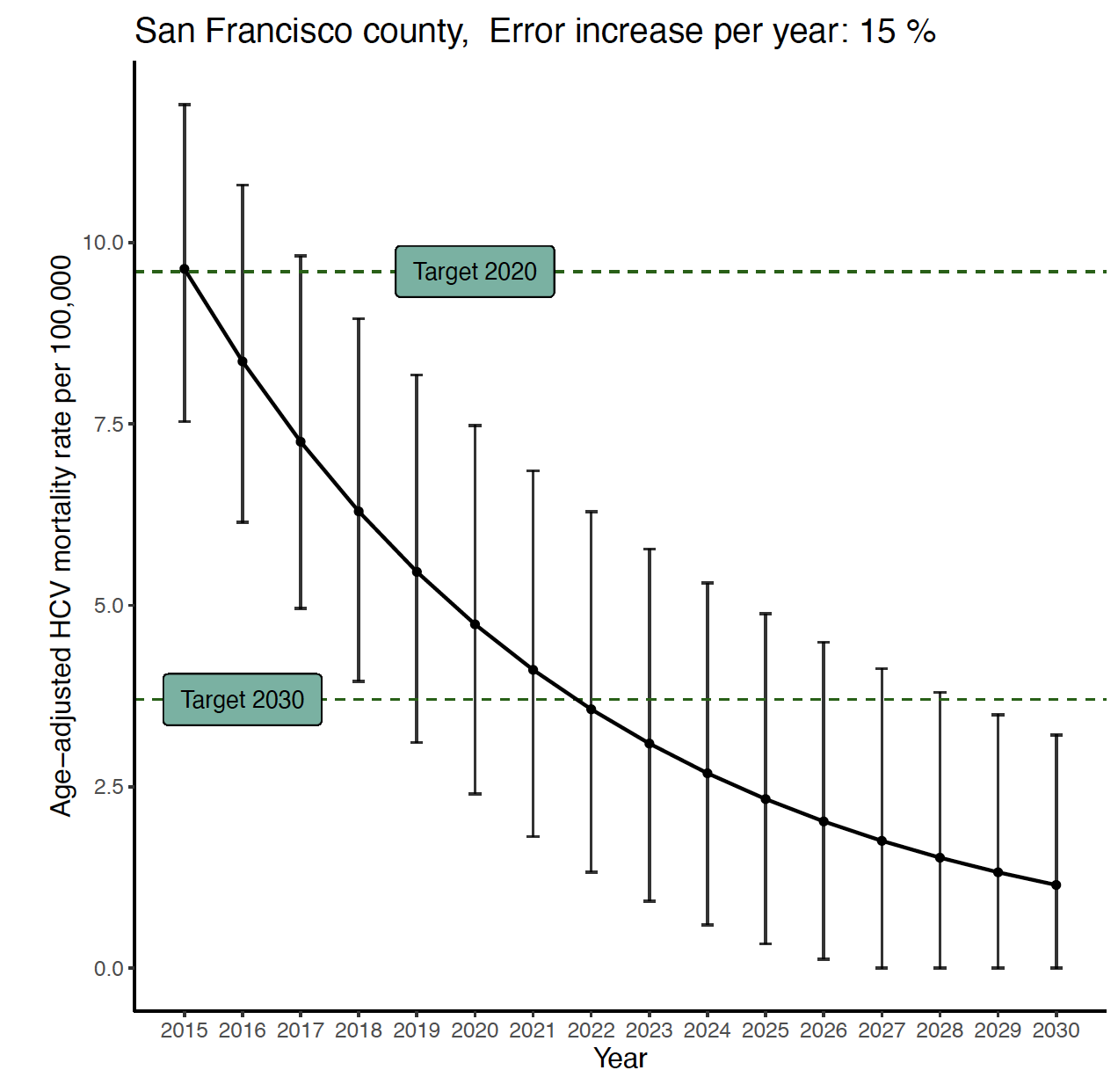 | 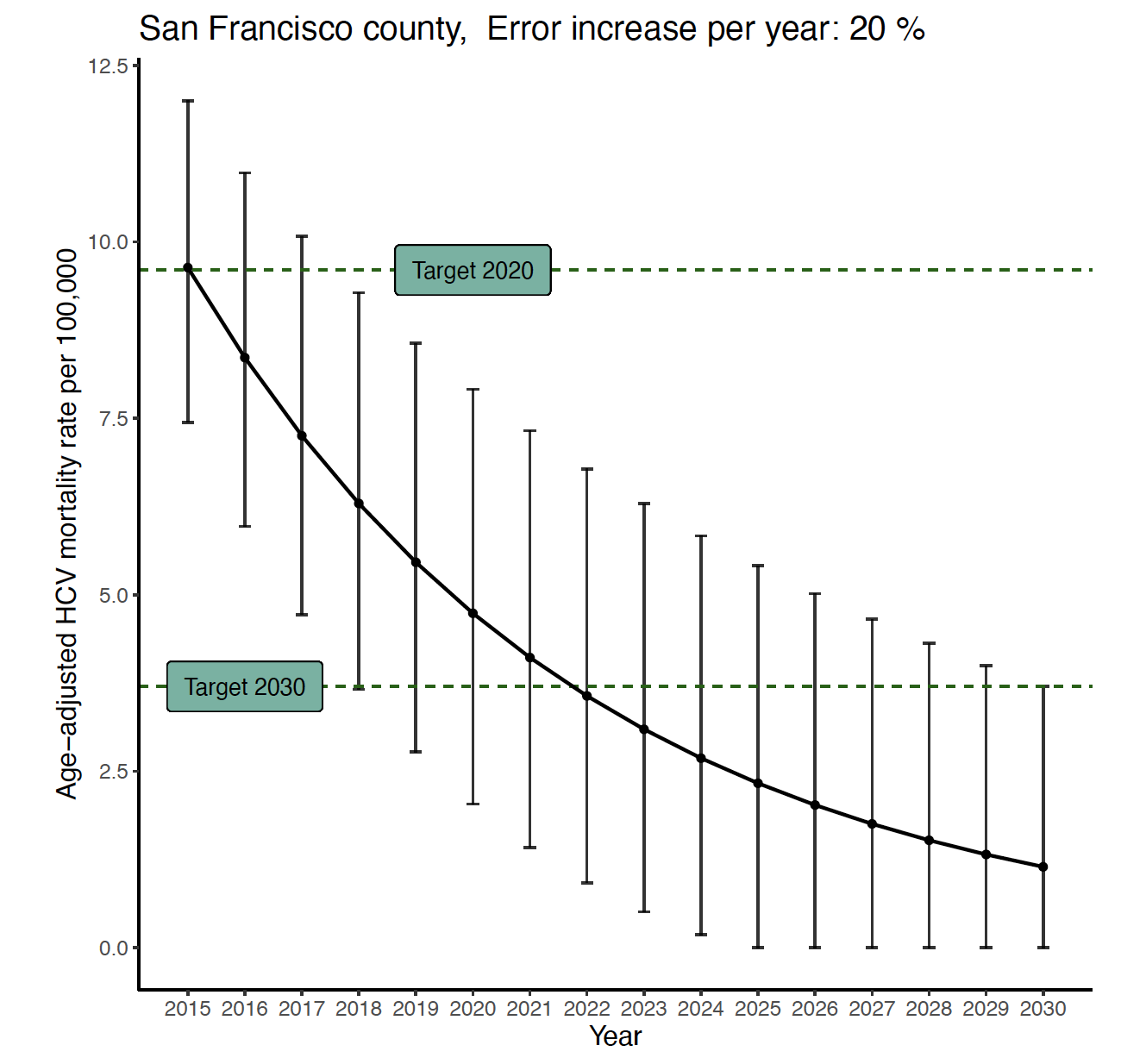 |
| 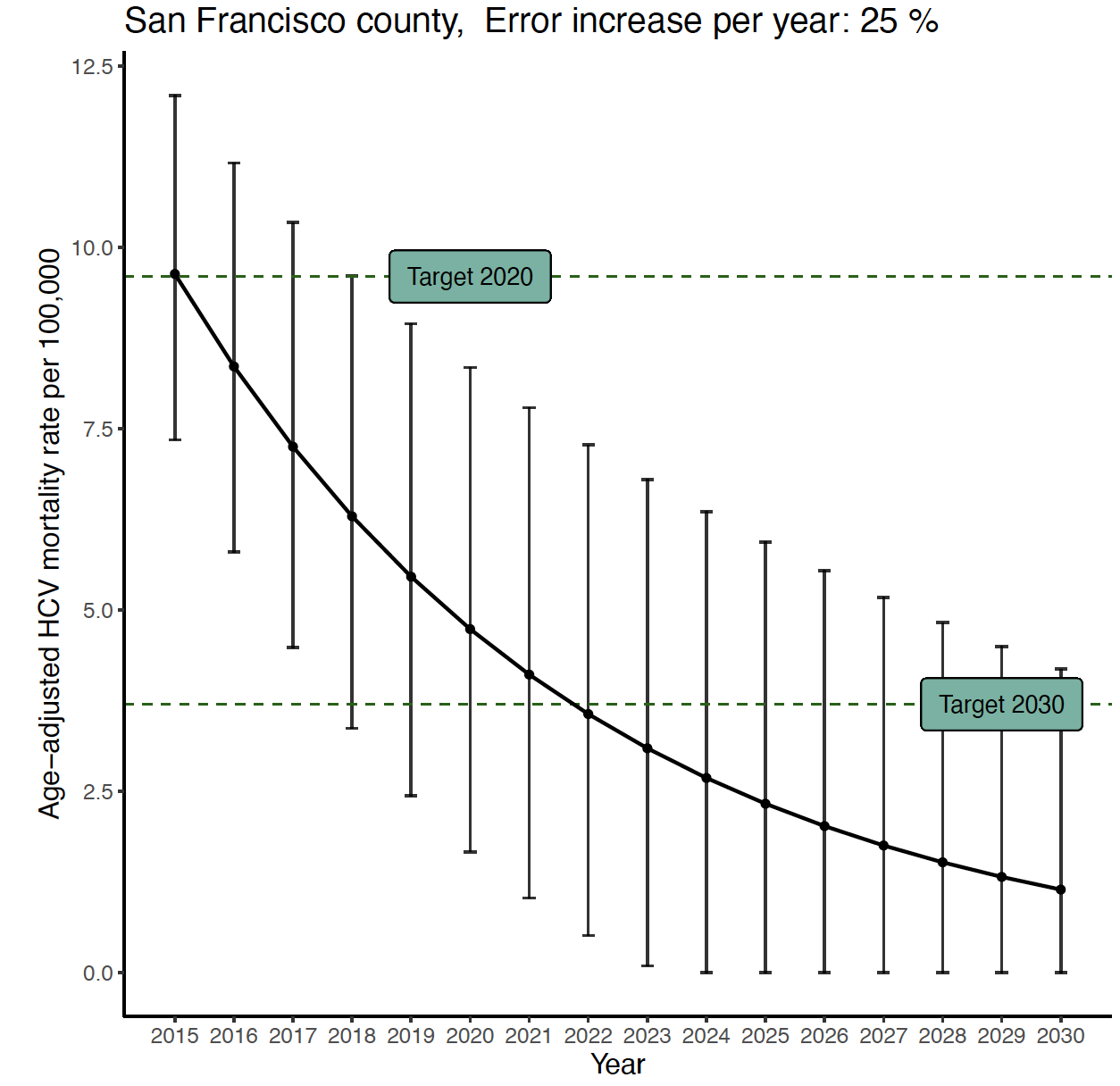 | 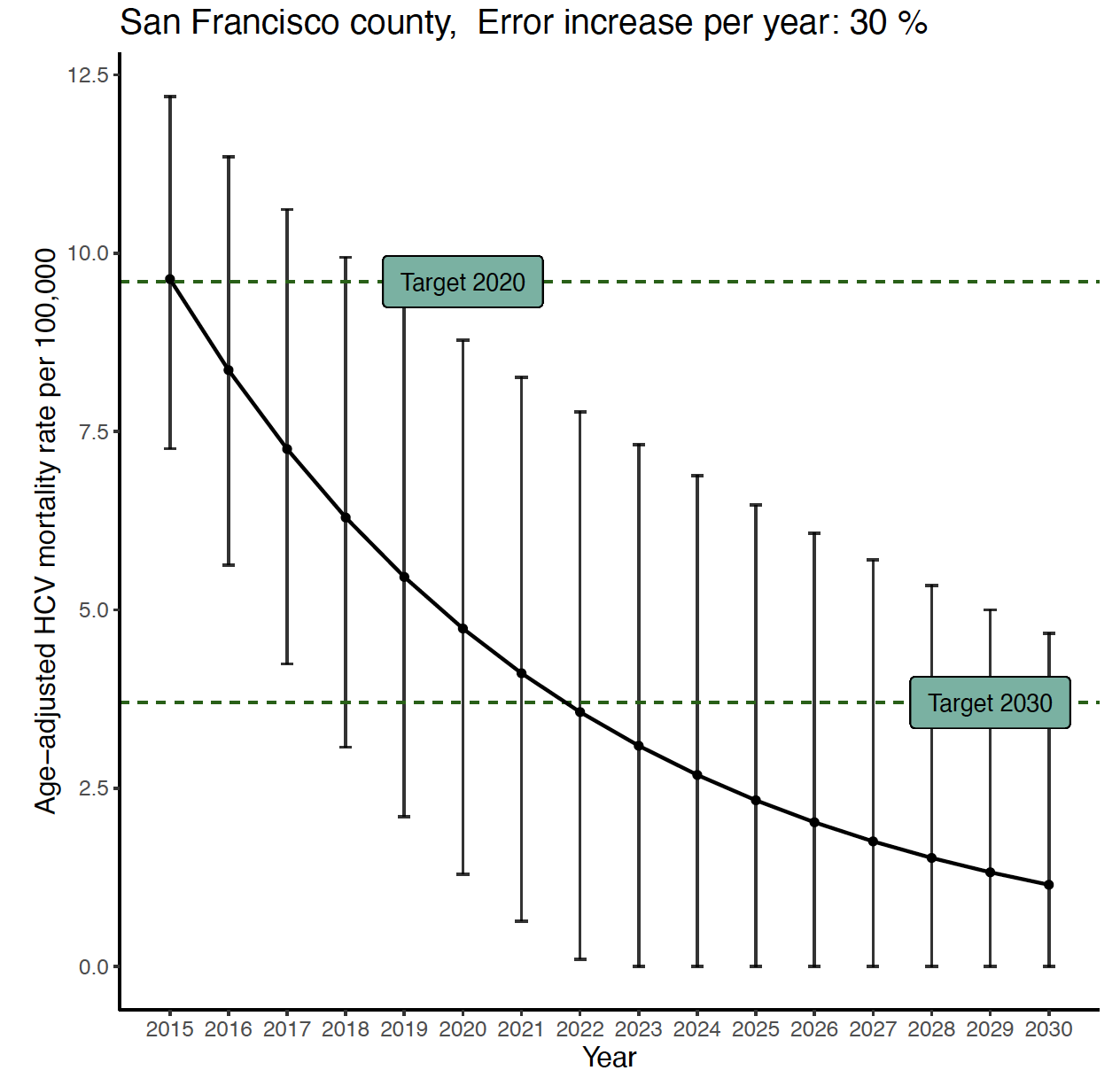 |

| **Supplemental figure 2.** Sensitivity analysis on future projection of HCV mortality for California (excluding San Francisco). The errors were increased per year for 0%, 5%, …, 30%. | 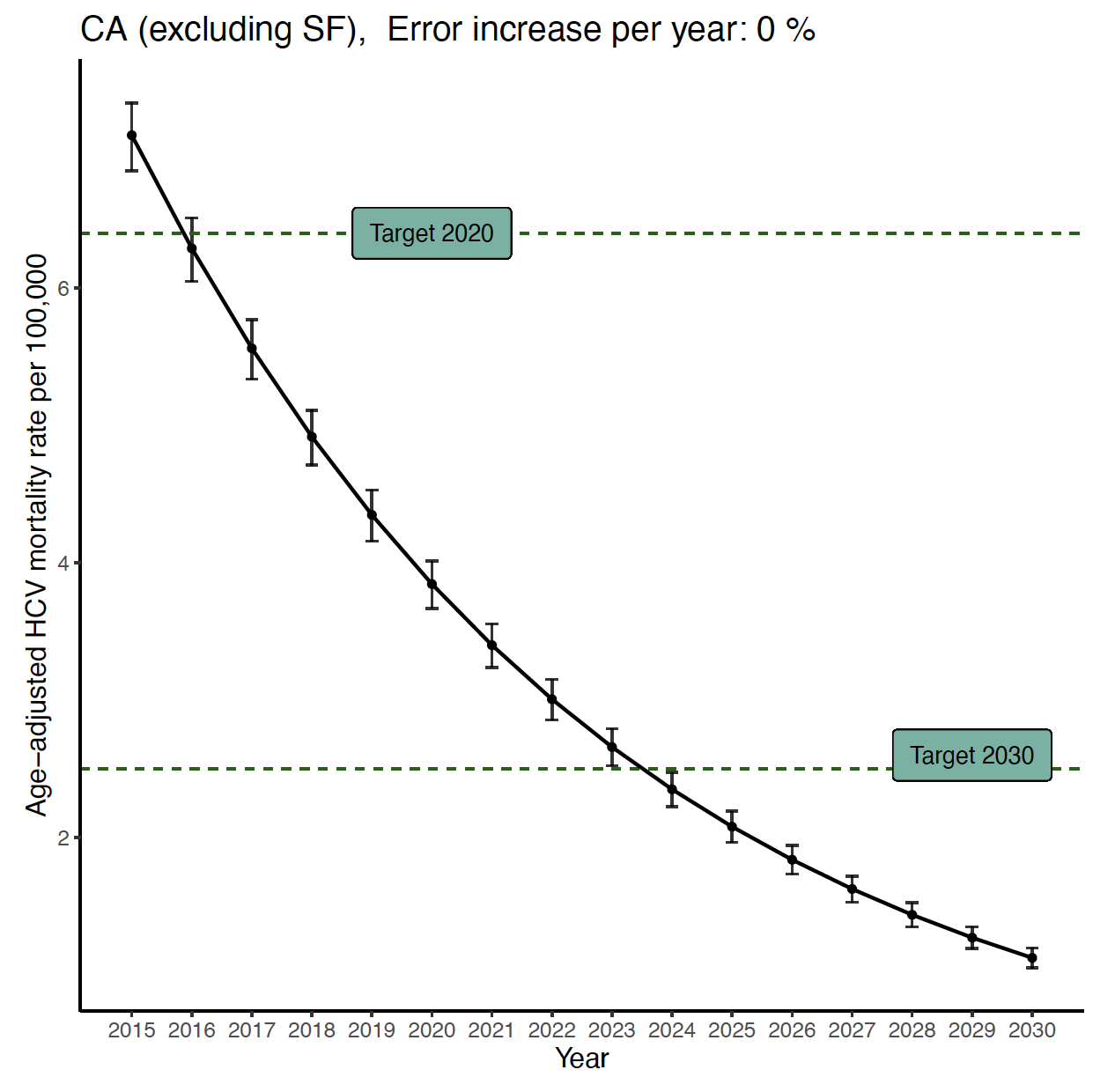 |
| --- | --- |
| 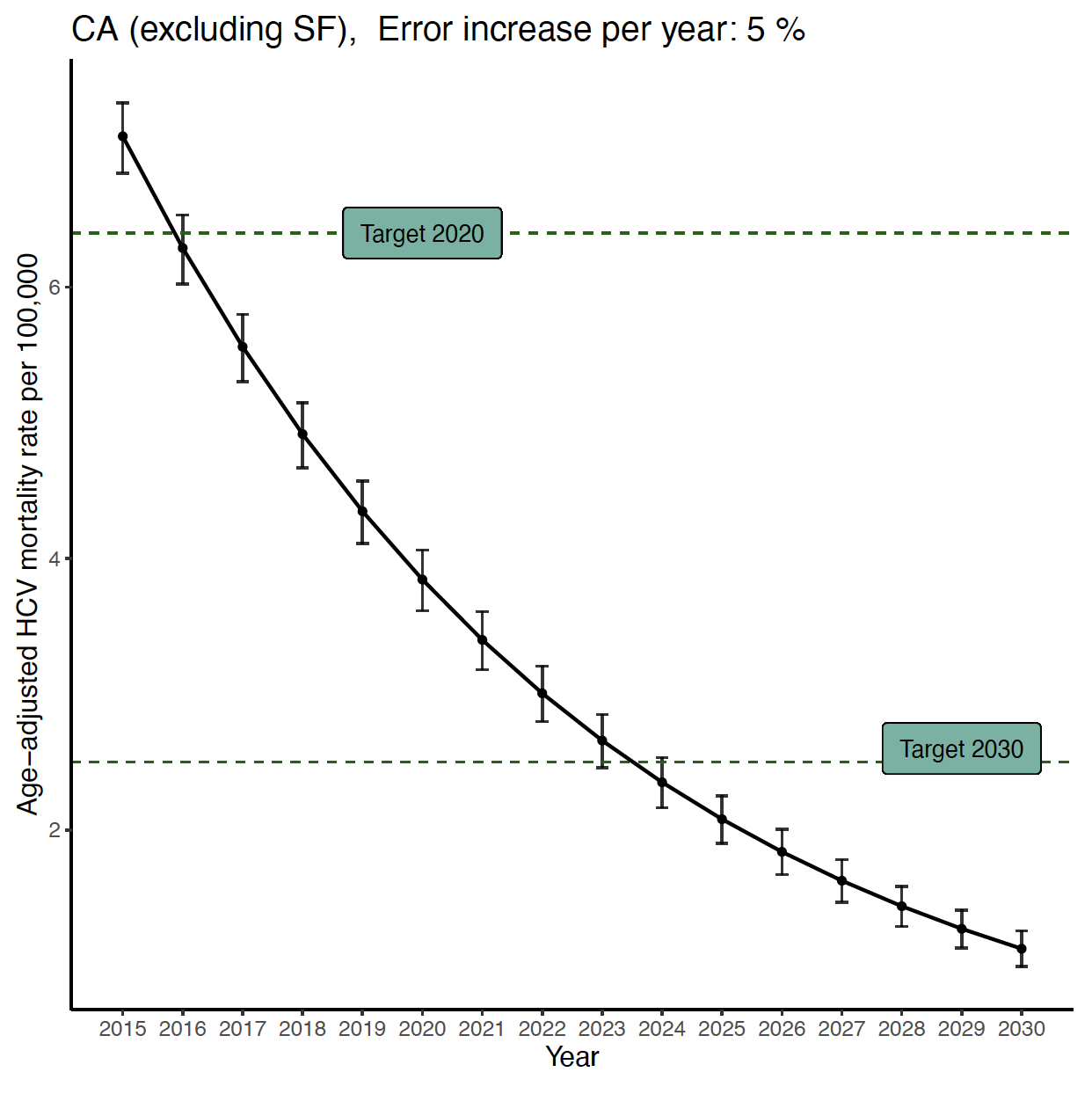 | 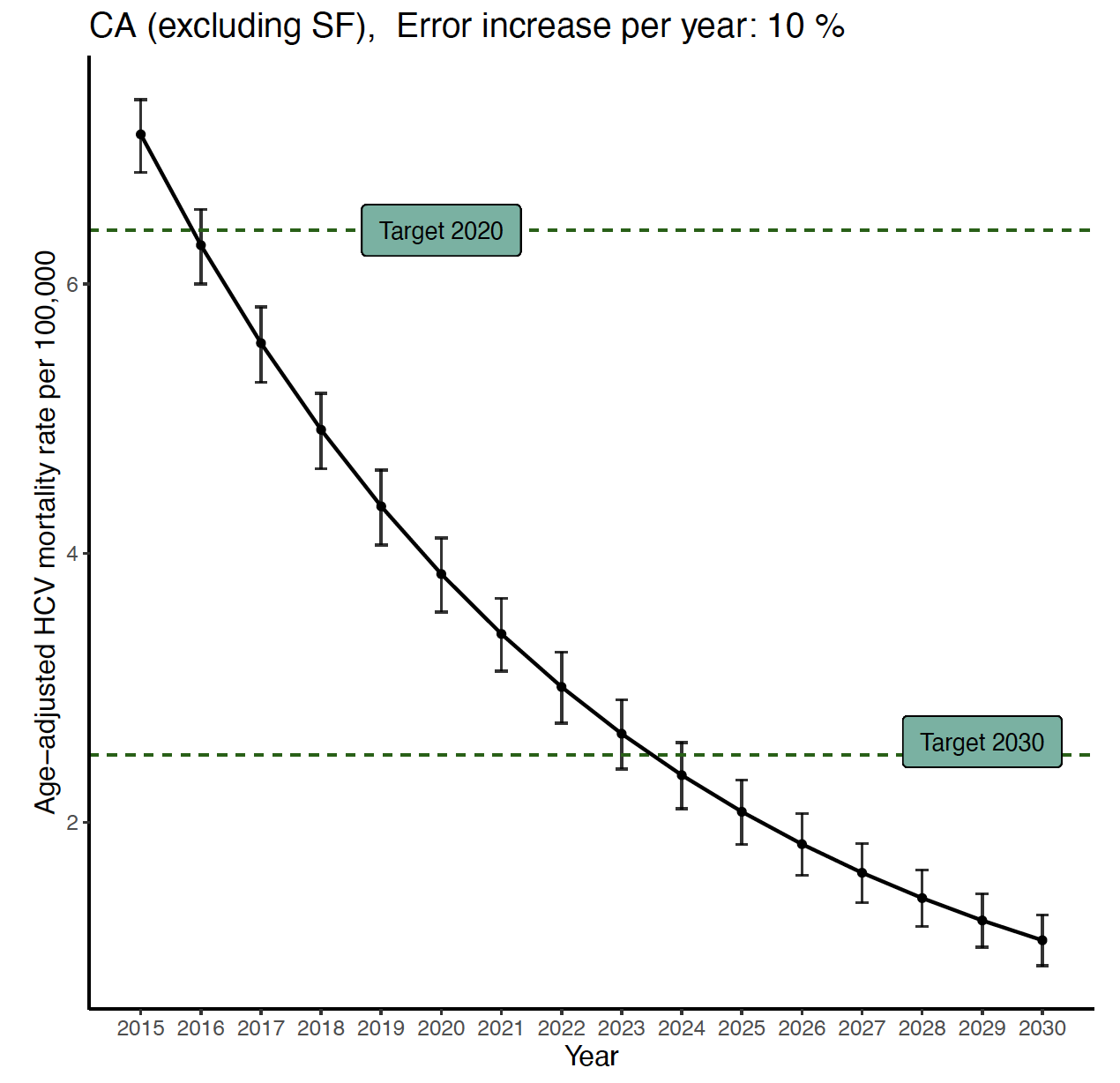 |
| 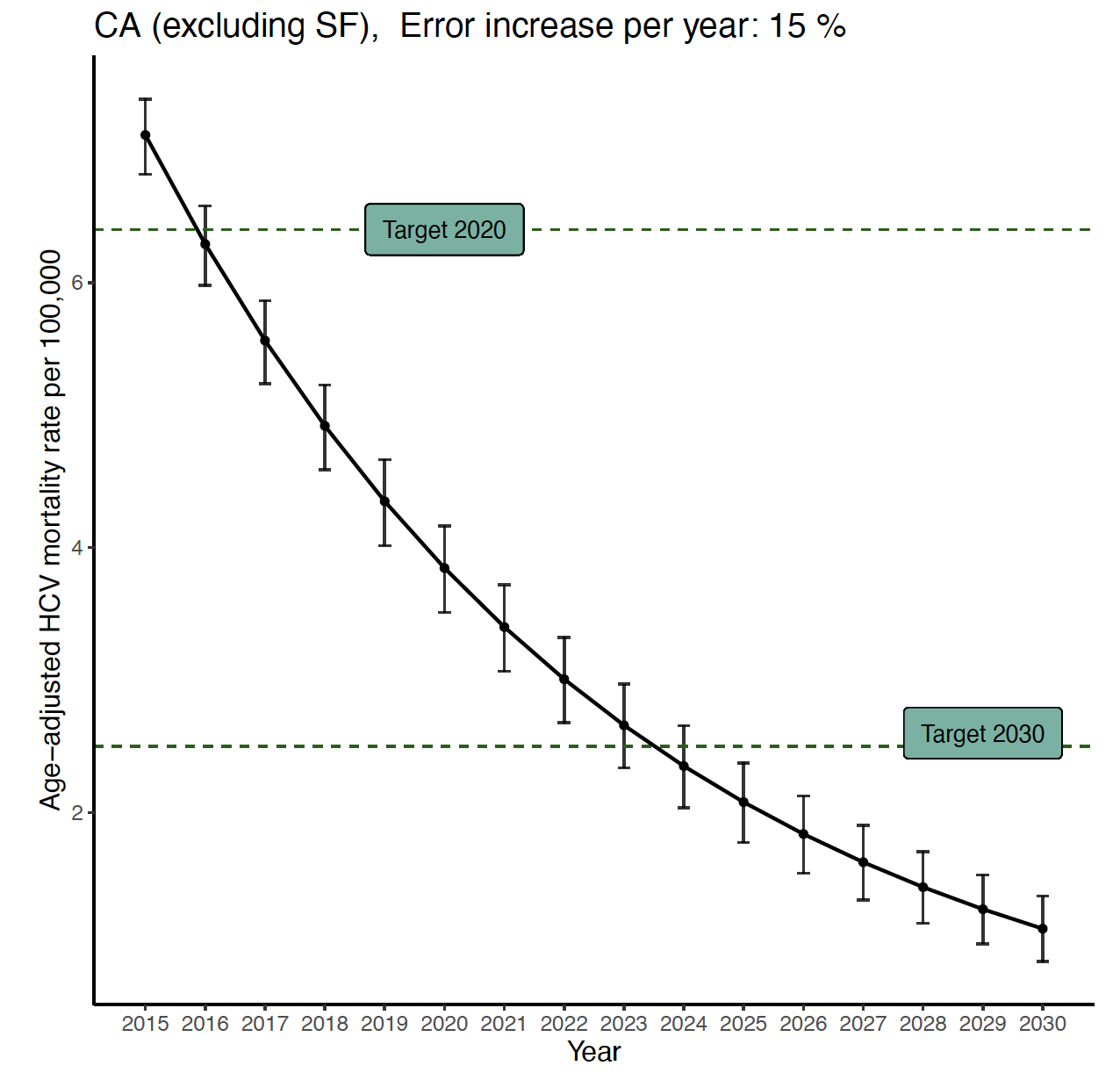 | 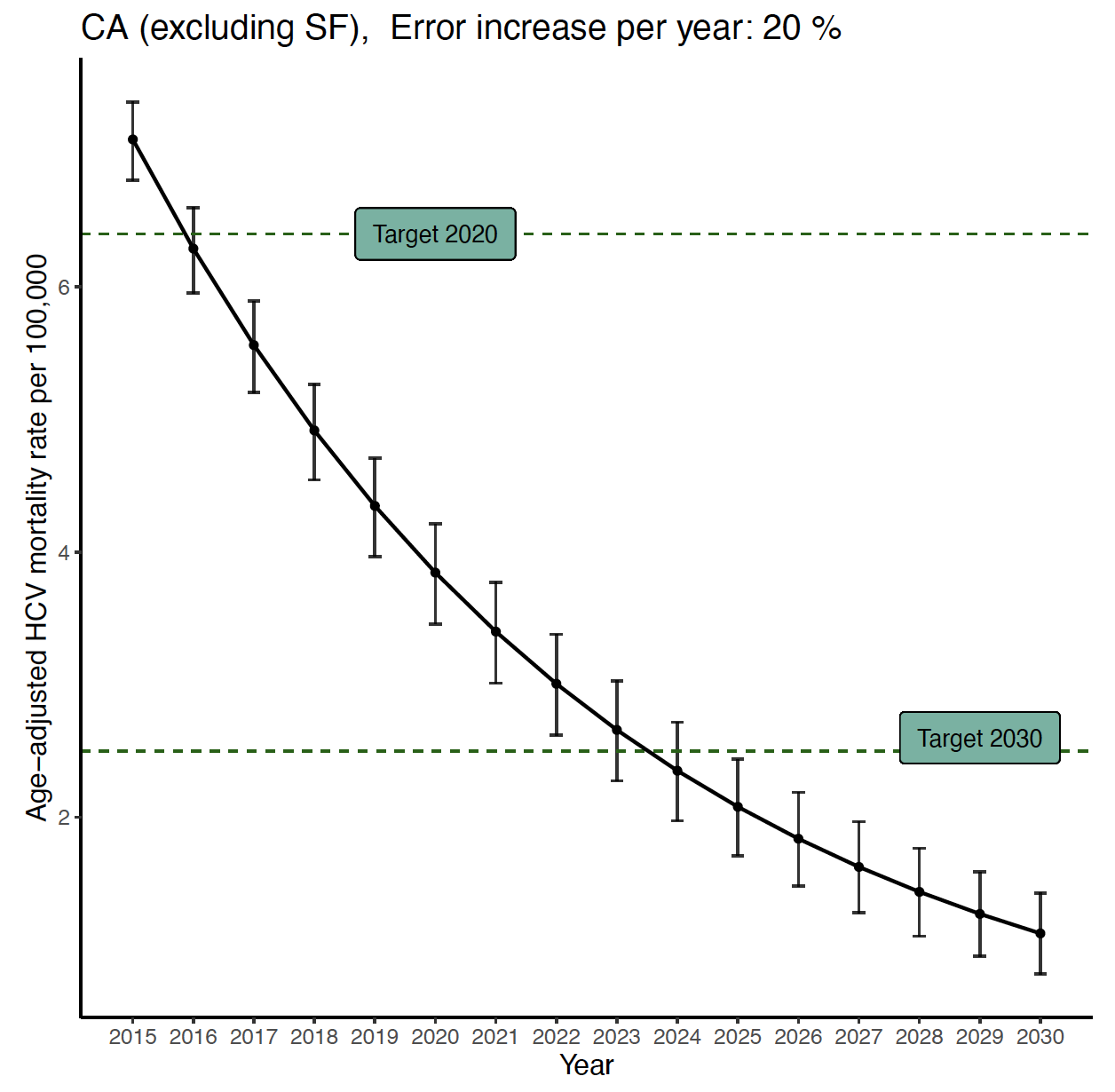 |
| 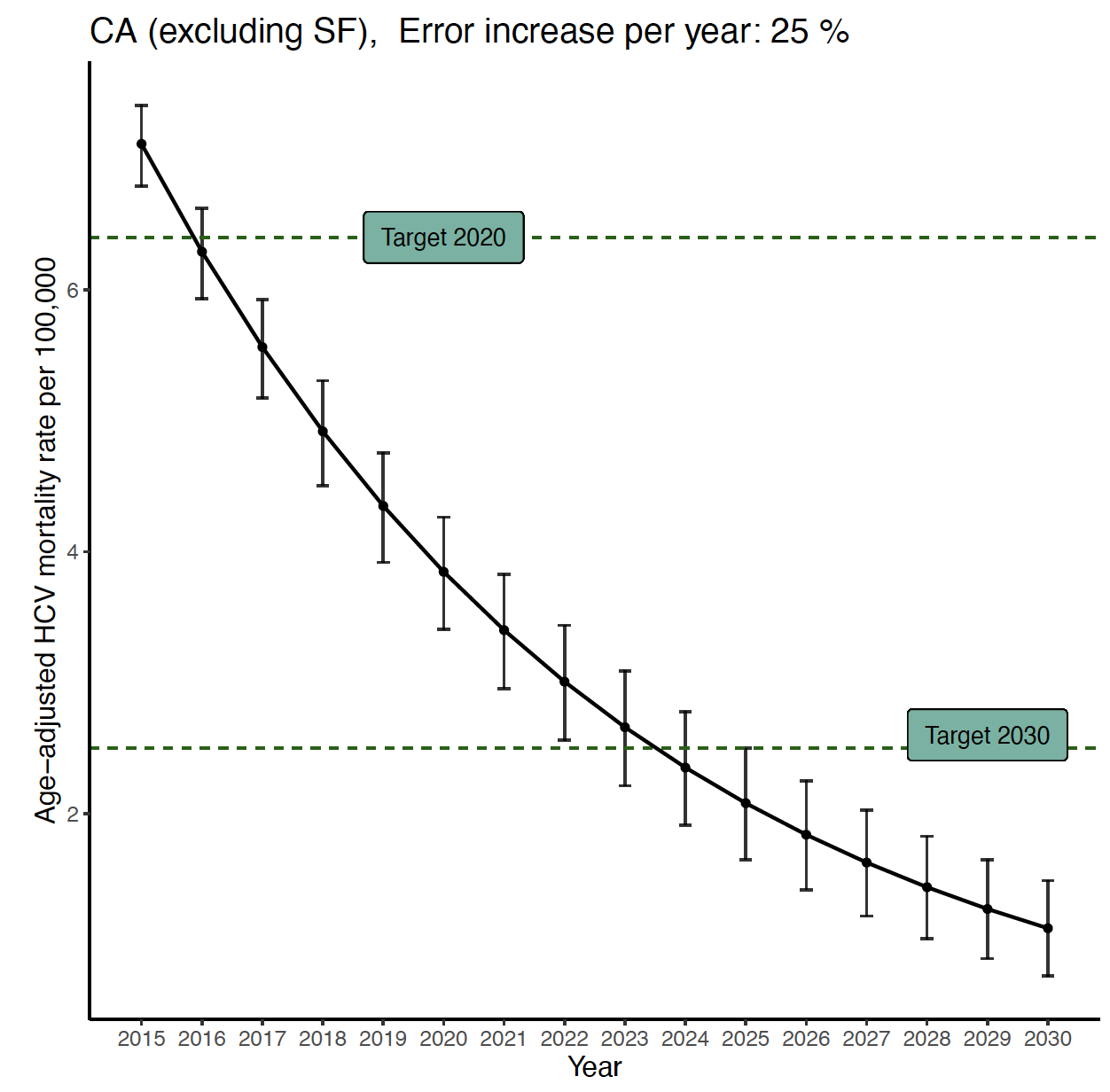 | 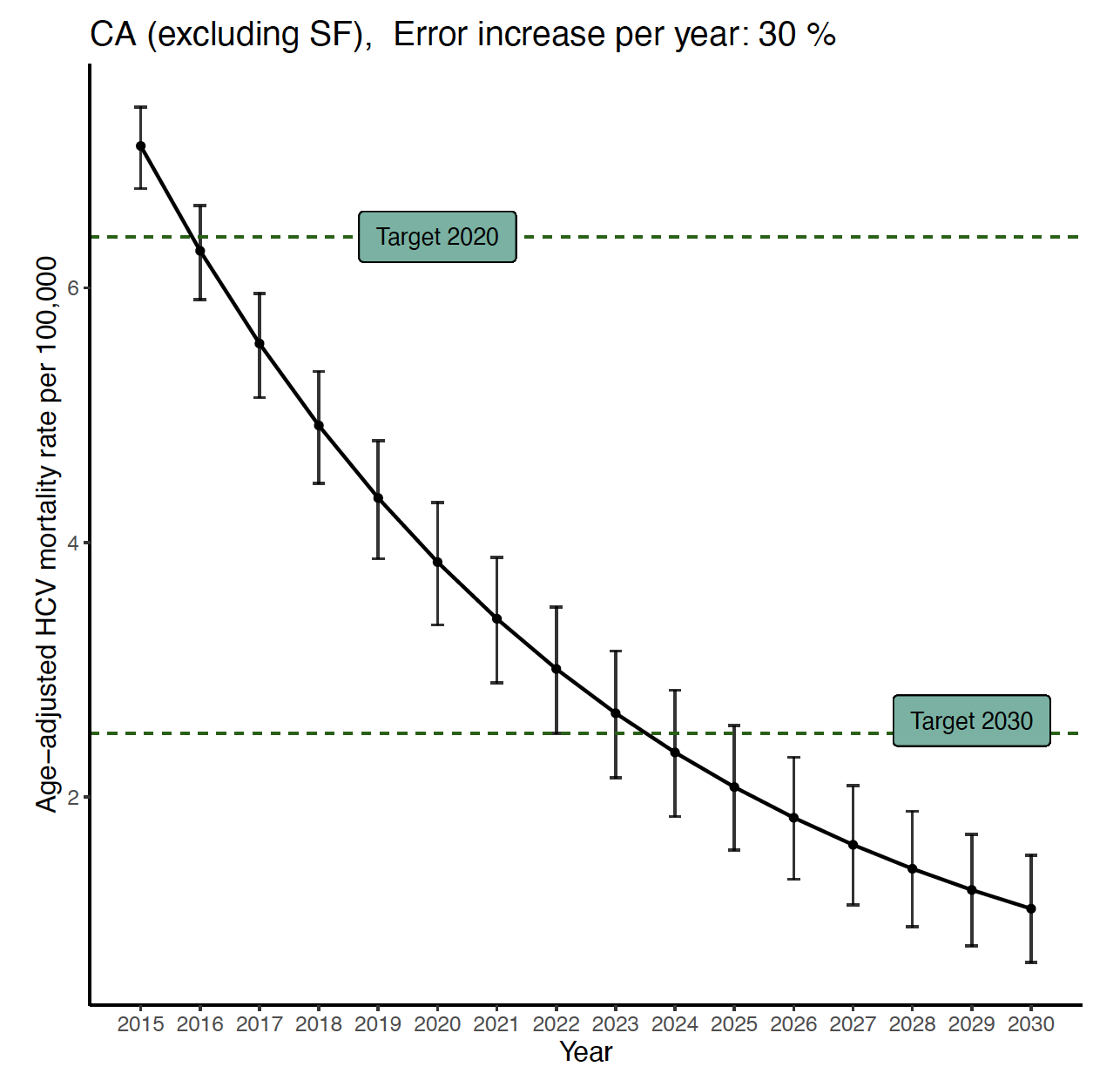 |

| **Supplemental figure 3.** Sensitivity analysis on future projection of HCV mortality for the United States (excluding San Francisco). The errors were increased per year for 0%, 5%, …, 30%. | 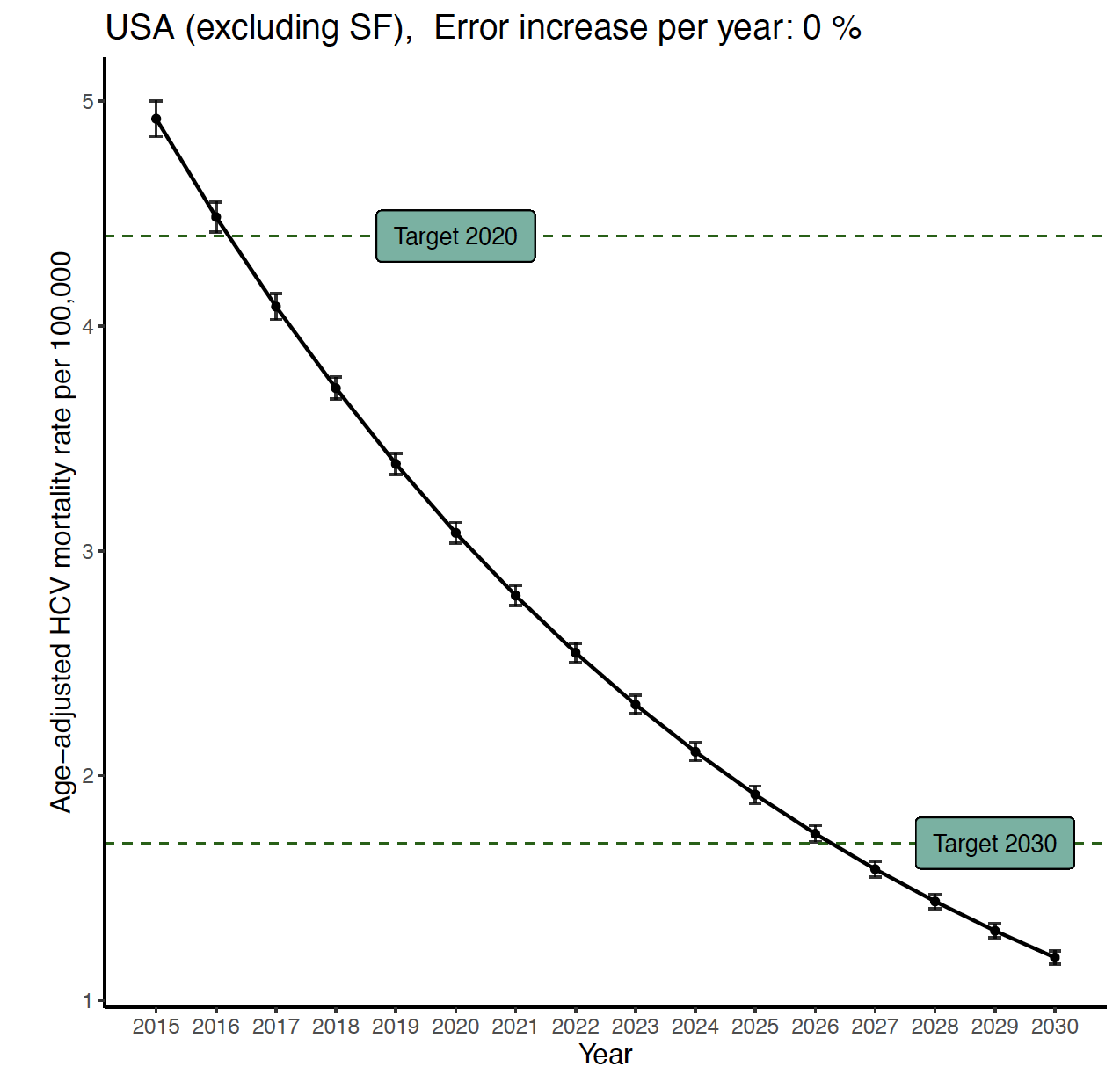 |
| --- | --- |
| 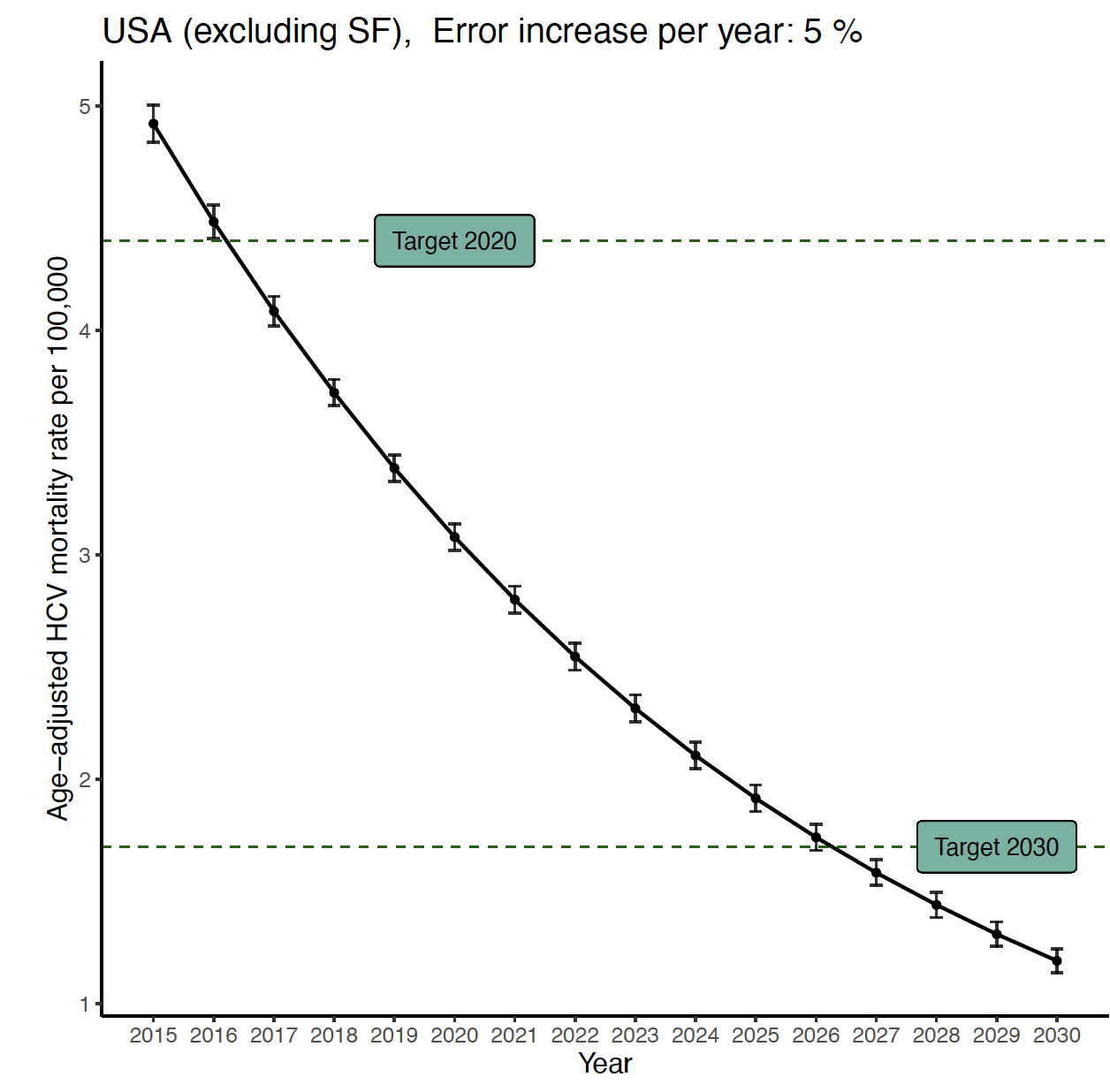 | 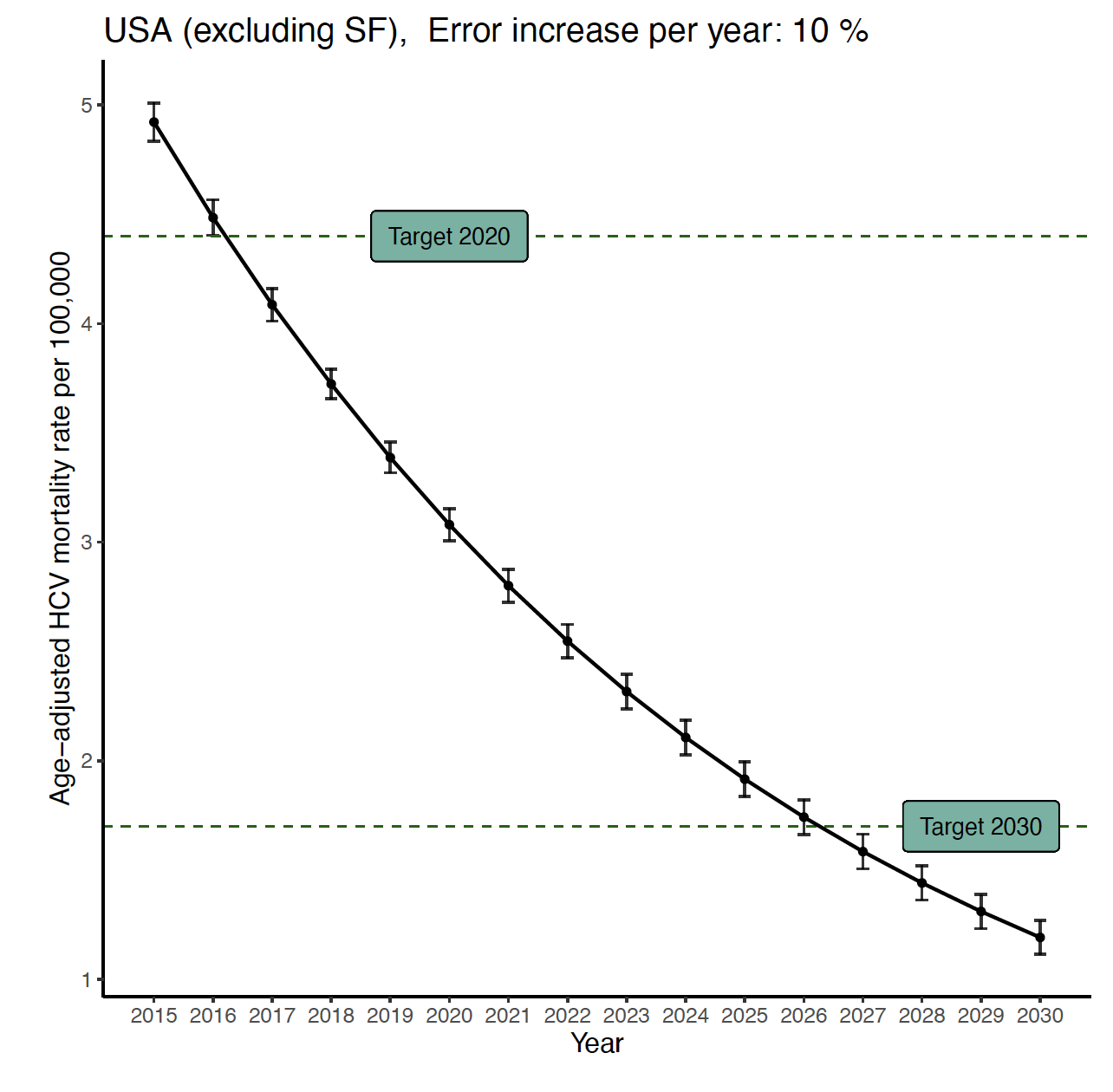 |
| 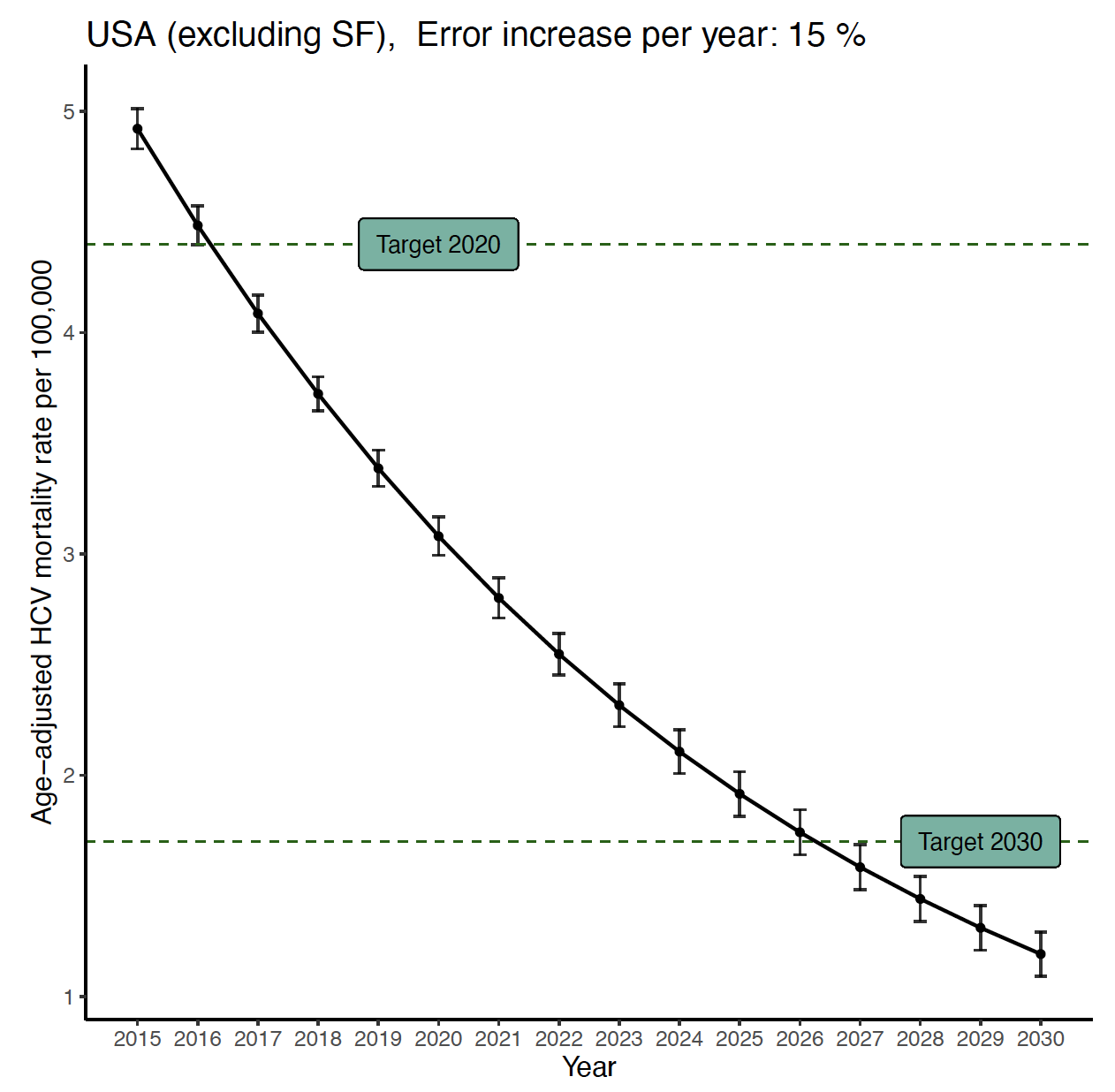 | 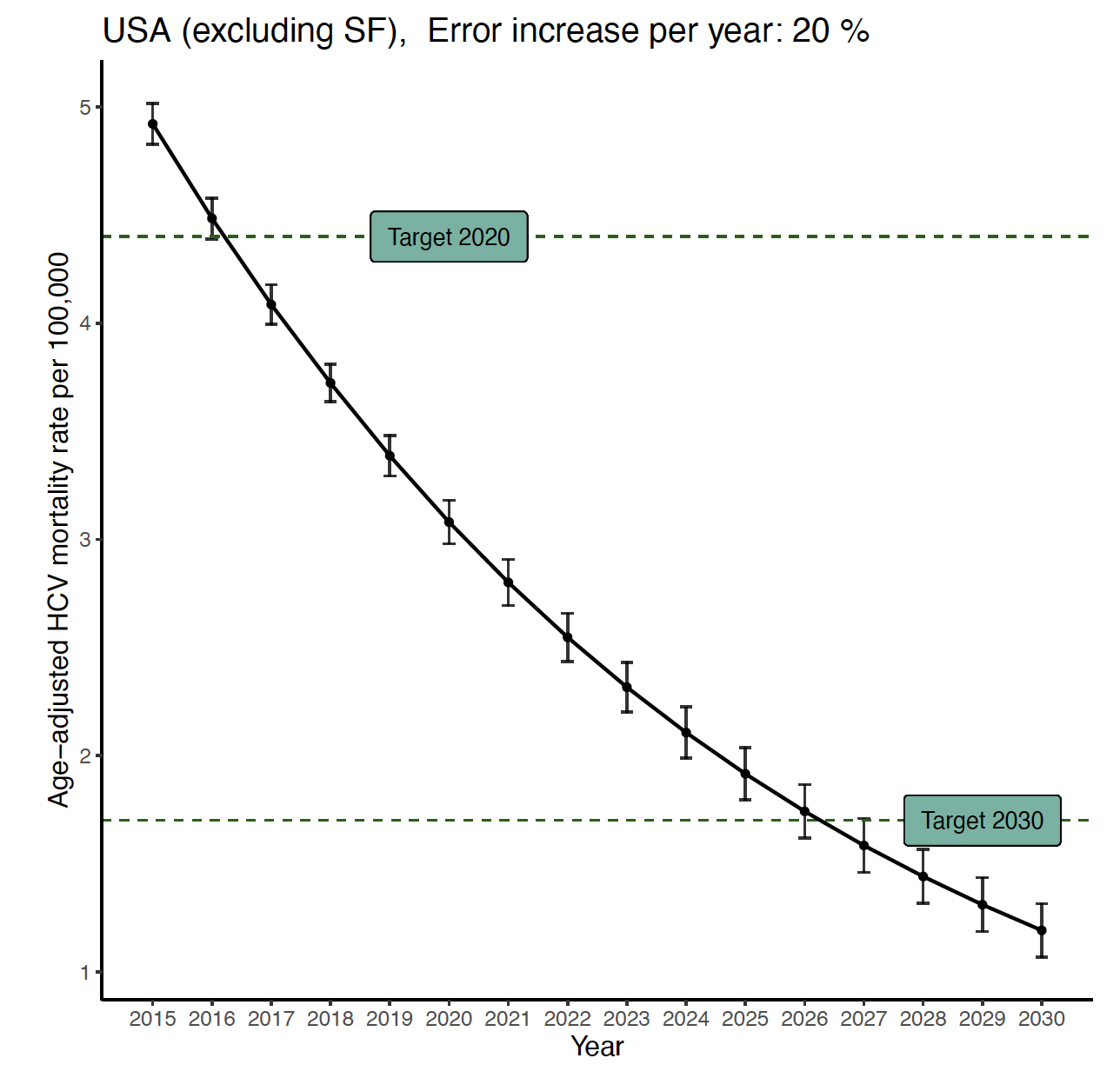 |
| 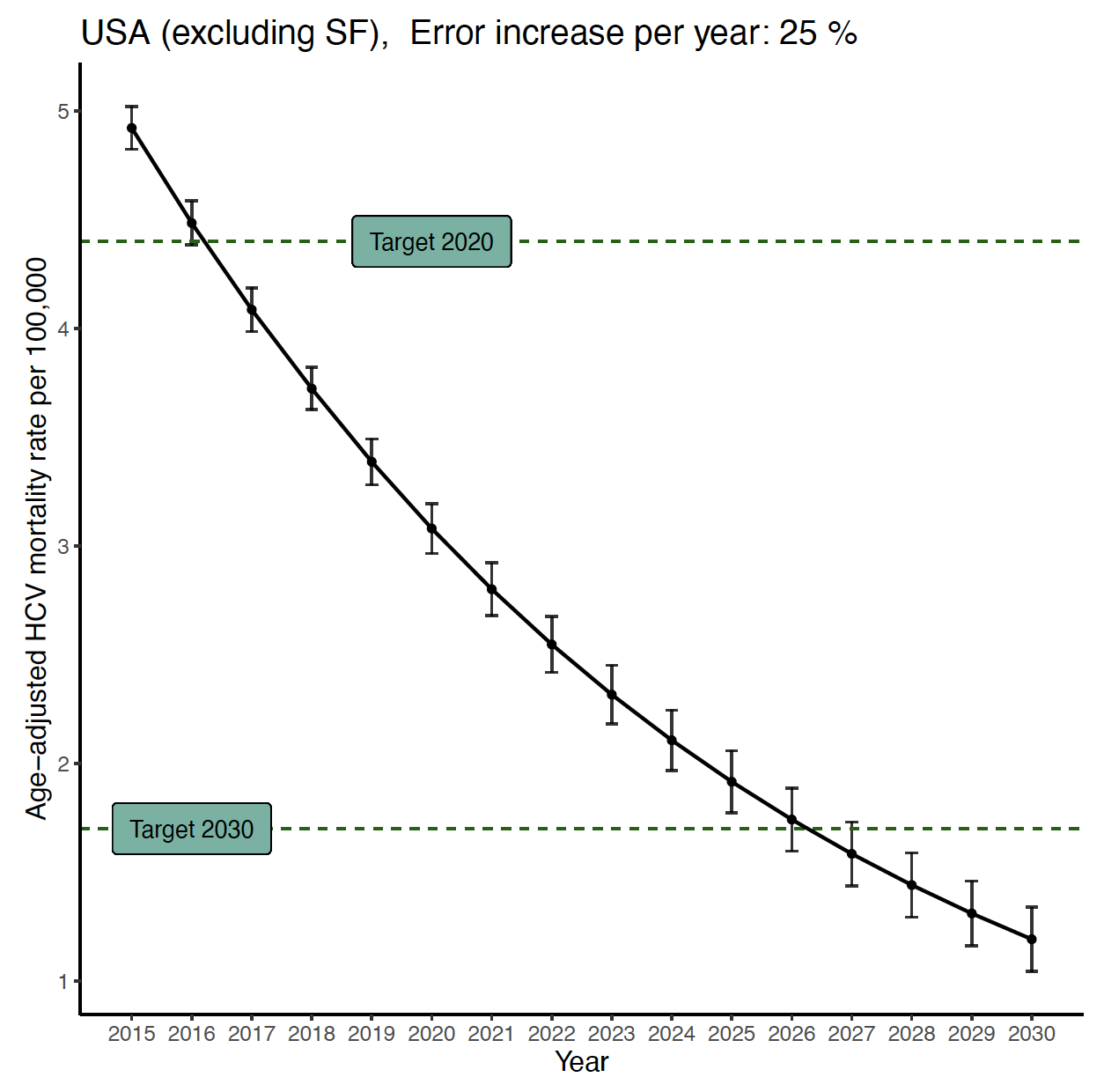 | 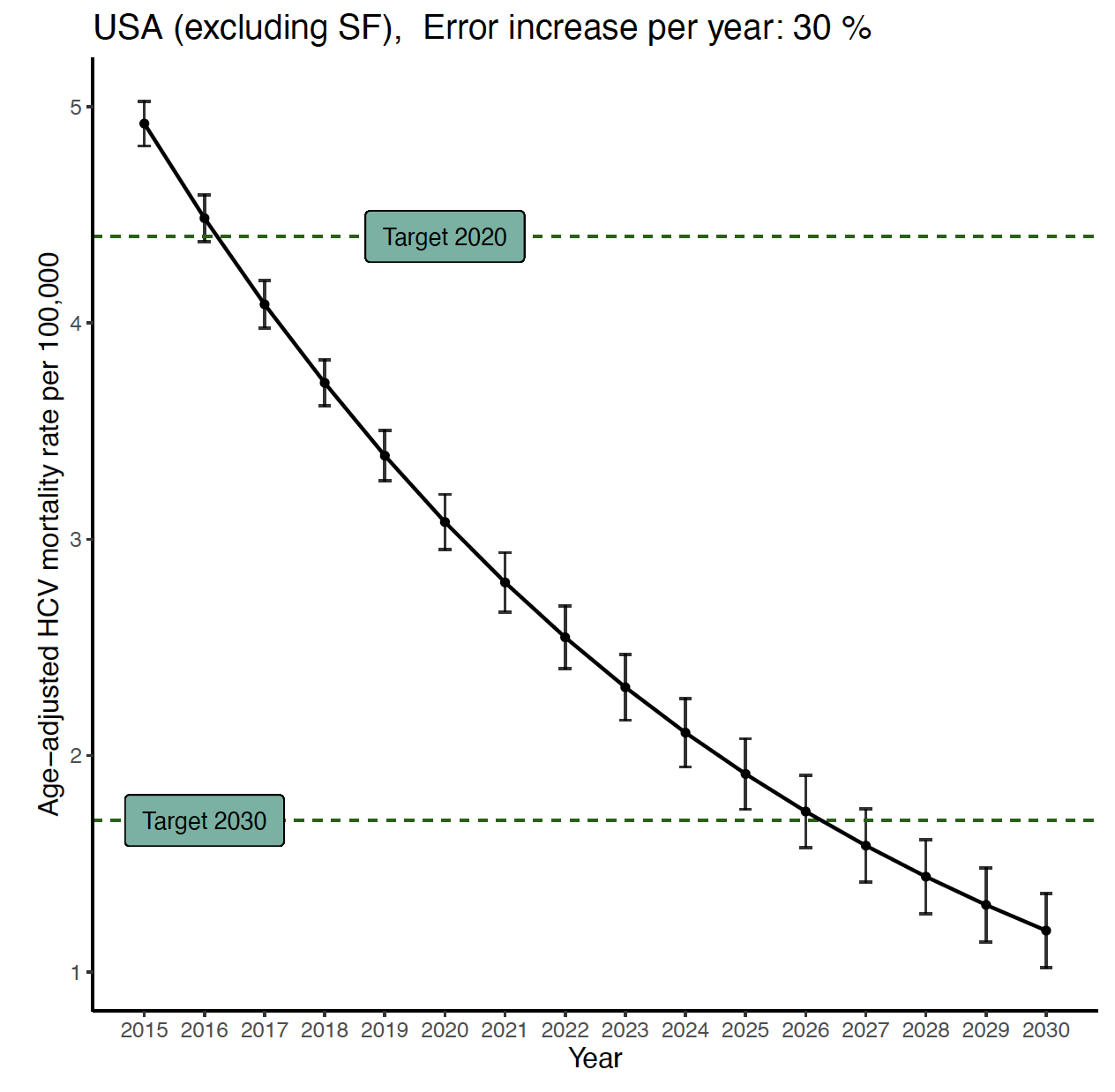 |
